# Unveiling nuclear localization signals in human arginine deiminase proteins

**DOI:** 10.1002/pro.70517

**Published:** 2026-03-02

**Authors:** José L. Neira, Olga Abian, Adrián Velazquez‐Campoy, Bruno Rizzuti

**Affiliations:** ^1^ IDIBE Universidad Miguel Hernández Elche Spain; ^2^ Instituto de Biocomputación y Física de Sistemas Complejos Universidad de Zaragoza Zaragoza Spain; ^3^ Instituto de Investigación Sanitaria Aragón (IIS Aragón) Zaragoza Spain; ^4^ Centro de Investigación Biomédica en Red en el Área Temática de Enfermedades Hepáticas y Digestivas (CIBERehd) Madrid Spain; ^5^ Departamento de Bioquímica y Biología Molecular y Celular Universidad de Zaragoza Zaragoza Spain; ^6^ CNR‐NANOTEC, SS Rende (CS), Department of Physics University of Calabria Rende Italy

**Keywords:** calorimetry, fluorescence, molecular docking, nuclear localization signal, PADI proteins, peptide

## Abstract

Arginine iminohydrolases are a family of enzymes involved in the conversion of arginine to citrulline. There are five isoforms in humans (PADI1, 2, 3, 4, and 6). Some of them are observed experimentally in the cytoplasm and in the nucleus of the cell; for moving to the latter location, they must pass through the cell nuclear membrane by using the translocation machinery, mainly formed by the proteins named importins. We have previously described and characterized the isolated PADI4 nuclear localization sequences (NLSs) and their binding to importin α3 (Impα3). By using theoretical predictors, here we foretold the existence of several NLSs in isoforms PADI1, PADI2, and PADI3. These predicted polypeptide regions were chemically synthesized, and the soluble ones were conformationally characterized in isolation. We studied their ability to bind Impα3 and its truncated species (ΔImpα3) without the importin binding domain, by using several biophysical techniques and molecular simulations. The isolated peptides were disordered and monomeric in solution. Moreover, all of them were capable of binding to both importin species with affinities in the low micromolar range, and targeting the canonical NLS binding site for cargo proteins. These findings suggest that the predicted NLS regions could be the sites for the binding of the corresponding intact PADI protein to importin, and therefore, any of the PADI enzymes could be translocated into the nucleus.

## 1 INTRODUCTION

Citrullination is a post‐translational modification catalyzed by L‐arginine iminohydrolases (PADIs), also known as peptidyl‐arginine deiminases (EC 3.5.3.15). PADIs intervene in apoptosis, tissue aging, nerve growth, inflammation, embryonic development, epithelial differentiation, and transcriptional regulation of gene expression (Asaga et al. 1998; Assohou‐Luty et al. 2014; Cuthbert et al. 2004; Ishigami and Maruyama 2010; Kizawa et al. 2008; Klose and Zhang 2007; Senshu et al. 1999; Wang et al. 2004). Furthermore, rheumatoid arthritis, psoriasis, Alzheimer's disease, multiple sclerosis, and many types of cancers are associated with an increased presence of any of the PADI isoforms and their citrullinated targets in different tissues (Anzilotti et al. 2010; György et al. 2006; Ishigami and Maruyama 2010; Zhu et al. 2023).

There are five human genes encoding PADI proteins: PADI1, PADI2, PADI3, PADI4, and PADI6 (Chavanas et al. 2004; Chavanas et al. 2008; Dong et al. 2006; Guerrin et al. 2003; Ishigami et al. 2002; Kanno et al. 2000; Nakashima et al. 1999; Zhu et al. 2023). Each isozyme is expressed differently in several tissues and organs: PADI1 is expressed in the uterus, epidermis, and in cancer cells, promoting cell migration, glycolysis, and cell invasion; PADI2 in secretory glands, brain, inflammatory cells, testis, and in several cancer cell types (Beato and Sharma 2020; Chen et al. 2017; Cherrington et al. 2012; Holmes et al. 2019; Horibata et al. 2017; Qu et al. 2018; Zhang et al. 2012); PADI3 in hair follicles and keratinocytes and in cancer cells; PADI4 in cancer cells, macrophages, monocytes, nuclear extracellular traps, and granulocytes; and PADI6 in embryos and oocytes during embryonic development (Liu et al. 2022; Qian et al. 2018; Zhang et al. 2016). Thus, as a common notable feature, the majority of the isoforms intervene in cancer‐related processes. For instance, PADI2 is involved in migration and progression of tumors, in angiogenesis, in cancer cell drug resistance, and even in promoting an increase of the number of extracellular vesicles. On the other hand, PADI3 can increase the invasiveness of tumor cells by mediating the formation of extracellular vesicles, that transport tumor‐promoting factors, and therefore, raising the invasiveness of tumor cells (Bednenko et al. 2003; Stewart 2007; Zhu et al. 2023). Finally, PADI1 has a key role in terminal differentiation of keratinocytes (Méchin et al. 2020) and it mainly acts as an oncogene in several cancers; moreover, citrullination of histones by PADI1 is key to early embryo development (Zhang et al. 2016).

All PADIs are found in the cytoplasm, but some of the above described functions suggest their presence within the nucleus. For instance, PADI4 has been detected in both the cytoplasm and the nucleus; and PADI2 is also found in the nucleus under some stress conditions (Cherrington et al. 2010; Cherrington et al. 2012; Horibata et al. 2017; Zhang et al. 2012). In the nucleus, both proteins citrullinate histones (Cherrington et al. 2010; Nakashima et al. 2002, as PADI1 does Zhang et al. 2016); these findings suggest that these proteins must be translocated through the nuclear pore complex (NPC). Nuclear translocation generally occurs through specific carrier proteins called importins, being helped by other auxiliary proteins (Bednenko et al. 2003; Stewart 2007). The most commonly described nuclear import pathway is activated by the recognition of a nuclear localization signal (NLS) polypeptide patch in the cargo (protein) by importin α (Stewart 2007). The complex formed between the cargo and importin α binds to importin β, and the ternary complex goes through the NPC. Importin α is a modular protein, having several α‐helix armadillo (ARM) repeat units (Bednenko et al. 2003; Stewart 2007). It contains three regions: (i) an N‐terminal importin β‐binding (IBB) domain, which mediates binding to importin β before transport through the NPC; (ii) a well‐folded NLS‐binding domain formed by 10 ARM units, which constitutes the main region of the protein; and (iii) a short and disordered C‐terminal region, whose exact function is unknown and which is often considered as a part of the main NLS‐binding domain. In the absence of importin β, the IBB binds to the ARM units involved in NLS recognition. This intramolecular interaction is relevant in cargo dissociation in the nucleoplasmic side (Kobe 1999).

We have previously studied the binding of the theoretically‐predicted, isolated NLS regions of PADI4 to Impα3 and to its truncated species lacking the IBB domain (ΔImpα3) (Neira et al. 2022). Binding of each isolated NLS region of PADI4 accounts for most of the binding of intact PADI4 to both importin species. We have considered Impα3 as a carrier due to its larger flexibility compared with other importins, allowing its interaction with various cargos (Smith et al. 2018). In addition, Impα3 can be considered a model protein to investigate how the NLS sequence of the protein can affect the thermodynamics of the binding, and we have already carried out several studies of the binding of Impα3 to other NLSs (Neira et al. 2020a; Neira et al. 2020b; Neira et al. 2021; Neira et al. 2022). Finally, by studying both importin species (with and without the IBB), we can explore whether the absence of the IBB domain affects binding of the isolated NLS region (Neira et al. 2020a; Neira et al. 2020b; Neira et al. 2021; Neira et al. 2022).

Because of the possible nuclear translocation of PADI1, PADI2, and PADI3 according to some of their functions, we tried to unveil their putative NLS regions (Kosugi et al. 2009a). First, we predicted potential NLSs in PADI1, PADI2, and PADI3 isozymes by using the well‐known cNLS Mapper predictor (Kosugi et al. 2009a; Kosugi et al. 2009b). And second, we tested the binding of those potential, isolated NLS regions to both Impα3 or ΔImpα3, by using the corresponding model peptides. It is important to stress out that, at the moment, we do not have any evidence that within the cell, nuclear translocation for those PADIs isozymes occurs exclusively through Impα3—for instance, PADI2 seems to be translocated upon Ca(II) stimulation by Ran GTPase (Zheng et al. 2019), another nucleocytoplasmic transporter, but rather, we are using Impα3 as a model in vitro for nuclear translocation.

A total of eight potential NLS polypeptide patches were predicted, and their corresponding peptides were synthesized and assayed. The soluble, isolated NLS regions were in all cases monomeric and disordered in solution. Different biophysical techniques confirmed that the four peptides—one from PADI1 and PADI2 and two from PADI3—were capable of binding to both importin species. Furthermore, docking simulations indicate that these peptides targeted specifically the NLS binding site of importin. Then, our in vitro and in silico results suggest that (i) PADI1, PADI2, and PADI3 contained putative NLSs and (ii) these NLSs were recognized by both Impα3 and ΔImpα3 at their corresponding binding site. As it has been already observed experimentally that PADI1 and PADI2 carry out some functions in the nucleus (Cherrington et al. 2010; Horibata et al. 2017; Zhang et al. 2012; Zhang et al. 2016), our results anticipate possible, and yet undiscovered, nuclear functions for PADI3.

## 2 RESULTS

### 2.1 Conformational features of the NLS peptides

Since some of the NLS peptides were not soluble (section 4.3) in aqueous solution at physiological pH—where both importin species have a stable native‐like structure (Díaz‐García et al. 2020)—we focused on the study of the conformational propensities and the interaction with both importin species of the soluble peptides. To address the characterization of the conformational features of the NLS peptides (Table 1), we carried out fluorescence, far‐UV CD, and NMR studies. The NLS regions within the whole structure of the three PADI proteins are shown in Figure S1, Supporting Information.

**TABLE 1:** Hydrodynamic properties of the NLS peptides derived from PADI1, PADI2, and PADI3.

NLS peptide^a^	MW (Da)	*D* (cm^2^ s^−1^) (*R*_h_, Å)^b^	*R*_h_, Å^c^
K^520^HQAKRSINEMLADRHLQRDNLHAQKSIDW^549^ (PADI1‐NLS1)	3697.16	(9.3 ± 0.1) × 10^−7^ (14 ± 3)	16 ± 2
K^499^LFREKQKDGHGEAIMFKGLGGMSSKRIT^527^Y (PADI2‐NLS2)	3455.00	(9.08 ± 0.09) × 10^−7^ (14 ± 2)	15 ± 2
H^361^KTLPVVFDSPRNGELQDFPYKRIL^385^ (PADI3‐NLS1)	3011.42	(9.10 ± 0.09) × 10^−7^ (14 ± 2)	14 ± 2
R^552^EVLKRELGLAESDIIDIPQLFKTERKKAT^581^ (PADI3‐NLS2)	3539.08	(9.4 ± 0.3) × 10^−7^ (14 ± 3)	16 ± 2

*Note*: All the peptides were amidated at their C‐termini, and acetylated at their N‐termini.

^a^ The numbering matches that of the intact, corresponding PADI isoform. The PADI1‐NLS1 peptide has the mutation Cys546Ser to avoid possible interchain disulfide formation and oxidation. The PADI3‐NLS2 peptide has the mutation Cys546Ser to avoid possible interchain disulfide formation and oxidation.

^b^ The *R*_h_ was determined by using the translational diffusion coefficient of dioxane, *R*_h_, of 2.12 Å (Wilkins et al. 1999) and the Stokes–Einstein equation. Errors in *D* are fitting errors to an exponential curve and errors in *R*_h_ are due to propagation errors in the calculation (Wilkins et al. 1999).

^c^ Calculated from the scale law: *R*_h_ = (0.027 ± 0.01) MW^(0.50±0.01)^ (Danielsson et al. 2002), where MW is the molecular weight of the peptide. Errors are due to propagation errors due to the equation used.

The fluorescence spectra of PADI1‐NLS1 showed a maximum at around 350 nm, which is characteristic of a solvent‐exposed tryptophan residue (Schmid 1997). On the other hand, the fluorescence spectra of PADI2‐NLS2 and PADI3‐NLS1 showed a maximum at ~308 nm, due to the additional tyrosine residue added at the C terminus; this value is characteristic of the fluorescence emission spectrum of a tyrosine, either in a buried or in a solvent‐exposed environment (Schmid 1997). Finally, the isolated PADI3‐NLS2 region did not show any fluorescence spectrum, as its sequence did not contain any tyrosine or tryptophan residue (Table 1): the sole aromatic residue was Phe573.

**FIGURE 1:**
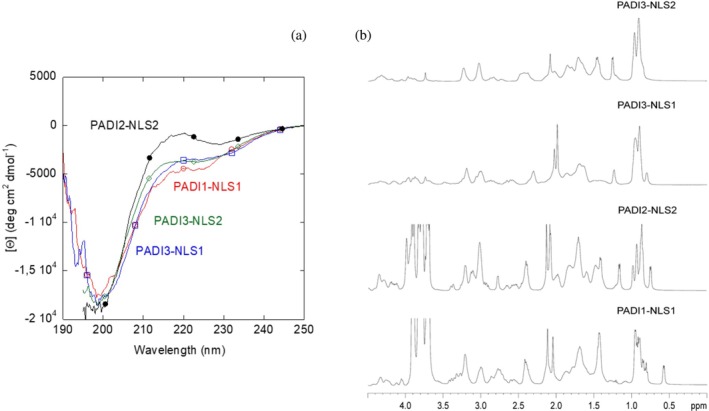
Conformational features of isolated NLS peptides in solution: (a) far‐UV CD spectra of NLS peptides at 25°C in sodium phosphate buffer (50 mM, pH 7.5). (b) Methyl region of the 1D‐^1^H‐NMR spectra of the isolated NLS peptides. The signal around 3.8 ppm, appearing in some of the spectra, corresponds to an impurity. Spectra were acquired at 10°C and pH 7.2 (50 mM, sodium phosphate buffer).

The far‐UV CD spectrum of each of the isolated NLS regions showed a minimum at ~200 nm (Figure 1a), indicating that the peptide had a random‐coil conformation (Kelly et al. 2005; Kelly and Price 2005; Woody 1995); however, there was a small shoulder for PADI1‐NLS1 at ~225 nm, which could be due to the presence of the aromatic Trp549 (Kelly et al. 2005; Kelly and Price 2005; Vuilleumier et al. 1993; Woody 1995). Although the far‐UV CD spectrum of PADI2‐NLS2 showed a minimum at ~200 nm (Figure 1a), there was a positive band at ~220 nm, which could be due to either the presence of a single C‐terminal tyrosine residue (Chakrabartty et al. 1993) or that of the other natural aromatic residues in the sequence, namely Phe501, His509, and Phe515 (Kelly et al. 2005; Kelly and Price 2005; Manning and Woody 1989; Woody 1995). In fact, PADI2‐NLS2 is, together with PADI3‐NLS1, the peptide with the largest percentage of aromatic residues in its sequence (including the additional non‐natural tyrosine at the C terminus; Table 1). The far‐UV CD spectra of PADI3‐NLS1 and PADI3‐NLS2 also showed minima at ~200 nm (Figure 1a), indicating that the conformations of both peptides were random‐coil (Kelly et al. 2005; Kelly and Price 2005; Woody 1995). PADI3‐NLS1 peptide has a large amount of natural aromatic residues, namely His361, Phe368, Phe379, and Tyr381, but its far‐UV CD spectrum did not show a positive band at ~220 nm as that of PADI2‐NLS2; this suggested that the presence of such a band in that spectrum was due to the location of the added C‐terminal tyrosine in PADI2‐NLS2 (Chakrabartty et al. 1993).

The disordered character of the NLS regions was further confirmed by the 1D‐^1^H‐NMR spectrum (Figures 1b and S2), which showed a clustering of the signals of all the amide protons between 8.0 and 8.6 ppm, whereas most of methyl protons were observed between 0.8 and 1.0 ppm. All these values are typical of protons belonging to disordered chains (Wüthrich 1986). Then, fluorescence, 1D‐^1^H‐NMR and far‐UV CD suggested that all peptides were disordered.

On the other hand, all the NLS peptides were monomeric, as concluded from: (i) the value of *D* measured by the DOSY experiment (Table 1); and (ii) the estimated *R*_h_ obtained from the comparison with the *D* of dioxane by using the Stokes–Einstein relationship (Wilkins et al. 1999). The estimated values of *R*_h_ were similar to those obtained theoretically for random‐coil polypeptides (Danielsson et al. 2002) with the same molecular weights (MWs) (Table 1).

**FIGURE 2:**
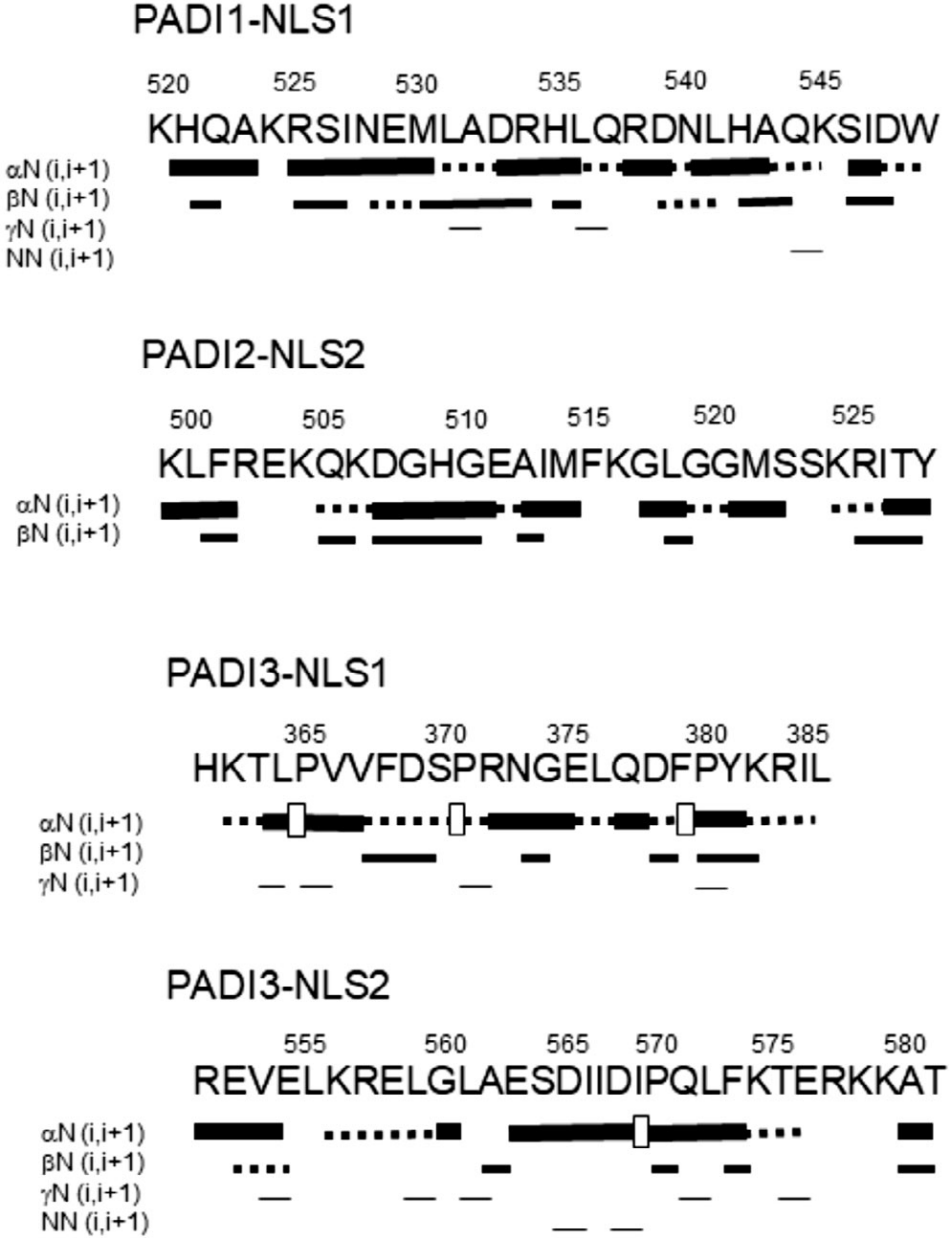
NOE diagrams of the isolated NLS peptides in aqueous solution from the 2D‐NMR NOESY spectra: NOEs are classified as strong, medium or weak, and represented by the height of the bar underneath the sequence; signal intensity was judged from the NOESY experiments. The dotted lines indicate NOE contacts that could not be unambiguously assigned due to signal overlapping. A white square indicates an αδ(*i*, *i* + 1) NOEs involving a proline residue.

To further confirm the disordered nature of the NLS peptides, we also carried out homonuclear 2D‐^1^H‐NMR experiments (Tables S1–S4). The peptides were mainly disordered in solution, as suggested by two pieces of evidence, further confirming the results from fluorescence, far‐UV CD (Figure 1a) and 1D‐^1^H‐NMR spectra (Figure 1b). First, the sequence‐corrected conformational shifts (Δ*δ*) of H_α_ protons (Kjaergaard et al. 2011; Kjaergaard and Poulsen 2011; Wüthrich 1986) for unambiguously assigned residues were within the commonly accepted range for random‐coil peptides (Δ*δ* ≤0.1 ppm) (Tables S1–S4). And second, no long‐ or medium‐range NOEs were detected, but only sequential ones (i.e., αN (*i*, *i* + 1) and βN (*i*, *i* + 1)) in the polypeptide patches fully assigned (Figure 2).

Therefore, we can conclude that all NLS peptides were monomers with a disordered conformation in isolation.

### 2.2 NLS peptides from PADI1, PADI2, and PADI3 could bind to Impα3 and ΔImpα3

To test whether the soluble peptides interacted with Impα3 and ΔImpα3 in vitro, we followed a two‐part experimental approach. First, we used steady‐state intrinsic fluorescence and far‐UV CD as spectroscopic techniques to observe a possible binding and concomitant conformational changes in the macromolecules; and secondly, we used intrinsic fluorescence and ITC to quantitatively measure the thermodynamic parameters of such binding.

**FIGURE 3:**
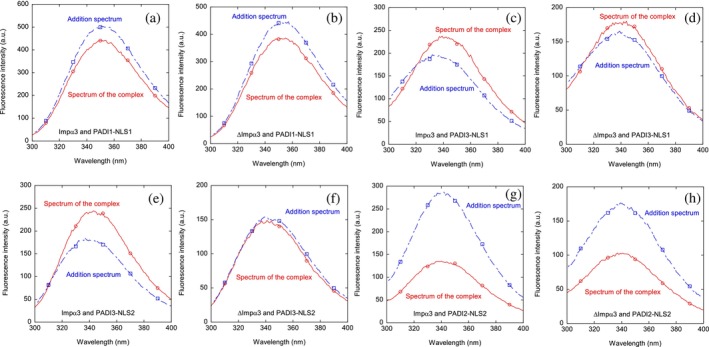
Binding of NLS peptides to either Impα3 or ΔImpα3 as monitored by fluorescence: fluorescence spectra of all complexes, obtained by excitation at 280 nm, of the NLS peptides with the importin species, and addition spectra obtained by the sum of the spectra of the two isolated corresponding macromolecules. The squares and circles, together with the continuous and dot‐and‐dashed lines, are drawn to guide the eye in each spectrum. All experiments were performed at 25°C.

We used fluorescence to determine whether there was a change in (i) the position of the maximum wavelength; (ii) the fluorescence intensity at such wavelength; or (iii) both of them, when the spectrum of the complex was compared to that obtained from the addition of the individual spectra of the two isolated polypeptides. A variation in fluorescence intensity by excitation at 280 nm was observed in all the spectra when the complex with Impα3 was formed with any of the NLS peptides (Figure 3), and in some cases, there were also changes in the maximum wavelength of the spectrum (as in the complex of Impα3 with PADI3‐NLS1); similar variations were observed by excitation at 295 nm for the complexes with Impα3 (where no fluorescence signal from PADI2‐NLS2, PADI3‐NLS1, or PADI3‐NLS2 should be observed, as they do not contain any tryptophan; Table 1). Furthermore, variations between the addition spectrum and that of the corresponding complex were also observed by excitation at both wavelengths (280 and 295 nm) when using ΔImpα3 to form the complex with any of the NLS peptides (Figure 3b,d).

**FIGURE 4:**
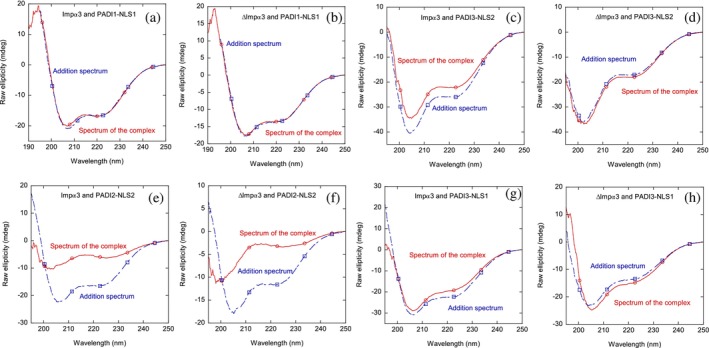
Binding of NLS peptides to either Impα3 or ΔImpα3 as monitored by far‐UV CD: The far‐UV CD spectra of selected complexes of several NLS peptides with the importin species, and addition spectra obtained by the sum of the spectra of the two isolated corresponding macromolecules. The squares and circles, together with the continuous and dot‐and‐dashed lines, are drawn to guide the eye in each spectrum. All experiments were performed at 25°C.

Next, we carried out far‐UV CD measurements, to confirm the fluorescence binding results. We observed three different trends characterizing the binding of the peptides. First, for PADI1‐NLS1, the far‐UV CD spectrum resulting from the addition of those of isolated polypeptides with either importin species was very similar to that of the complexes, in the case of both importin species (Figure 4a,b). These results do not mean that binding does not occur, but rather that if there is binding, the reaction does not alter the conformational preferences of any of the two polypeptides (and probably those of the aromatics). In fact, it is with the joint use of fluorescence and far‐UV CD spectra that we can get reliable conclusions on the possible binding. In the second observed tendency, we take as an example, the behavior of PADI3‐NLS2 (Figure 4c) with Impα3, where the far‐UV CD addition spectra showed changes; these changes were smaller when ΔImpα3 was used to form the complexes; the same behavior was observed with PADI3‐NLS1. And finally, for PADI2‐NLS2 and its complexes with both importins, the changes between the addition spectrum and those of the complex were very important (Figure 4d).

Then, taken together, the steady‐state fluorescence and far‐UV CD spectra allowed us to conclude that there was binding of each of the four peptides to each of the importin species.

Next, we determined quantitatively the affinity of the NLS peptides for each of the importin species. To that end, we used three biophysical techniques. We carried out fluorescence titrations to determine the affinity constant, fixing the concentration of each of the importin species, and raising that of the corresponding peptide. We also used ITC to determine, together with the affinity constant, the values of the enthalpy and the entropy of the binding reaction. And, finally, we used BLI to determine the kinetic parameters of the binding reaction and to estimate an affinity constant (to compare with the other two techniques) of the reaction, assuming a two‐state process for the binding of each peptide to the importin species. It is important to pinpoint that, in the context of the fluorescence experiments, we are also assuming that there were only significantly populated the unbound and the free species (Equation (1)). However, in the context of the BLI experiments, if we assume that there are more than the two species (i.e., an intermediate one between the free and bound states) the equilibrium dissociation constant cannot be obtained as: *K*_d_ = *k*_off_/*k*_on_, but rather by using more complex relationships (Fersht 2017).

**FIGURE 5:**
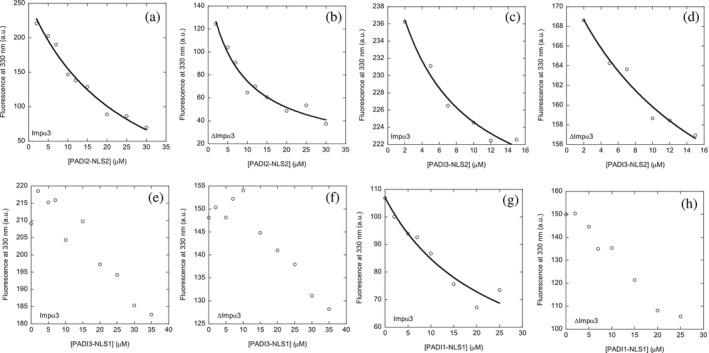
Quantitative determination of the binding of all NLS peptides to either Impα3 or ΔImpα3 as monitored by fluorescence: The curves through the data are from the fittings to Equation (1). Experiments were carried out at 25°C.

We first carried out fluorescence titrations with each of the four peptides and either Impα3 or ΔImpα3. In all cases, we observed a decrease of the fluorescence intensity as the concentration of the corresponding peptide was increased (either by following the titration at 280 or 295 nm); however, under some conditions, such variation could not be fitted to Equation (1) (Table 2) and, in those cases, we were not able to determine a reliable *K*_d_ value (Figure 5). The impossibility of obtaining trustworthy titration curves was probably due to the fact that the affinity constant value was beyond the range accessible, at least at the concentrations we used for the NLS peptides and/or importin‐species in our experiments (Royer and Scarlata 2008) (Figure 5). Although we could not determine consistent *K*_d_ values for some peptides, our results suggest that, since most of the designed peptides contained tyrosine or phenylalanine residues (Table 1), the binding involved at least some of the tryptophans of the importin species (six in each species). Only for PADI1‐NSL1, which contained a tryptophan residue in its sequence (Table 1), we could not ascertain the involvement of the tryptophans of Impα3 and ΔImpα3 in peptide binding.

**TABLE 2:** Affinity constants of the NLS peptides determined by fluorescence titrations at 25°C.

NLS peptides	Impα3	ΔImpα3
	*K*_d_ (μM)	*K*_d_ (μM)
PADI1‐NLS1	15 ± 12	–^a^
PADI2‐NLS2	22 ± 9	8 ± 2
PADI3‐NLS1	–^a^	–^a^
PADI3‐NLS2	6 ± 3	16 ± 12

^a^ A decrease in fluorescence intensity was observed as the corresponding peptide concentration was raised, but no trustable *K*_d_ values could be obtained from data fitting to Equation (1). Indicated errors are fitting errors to Equation (1).

**FIGURE 6:**
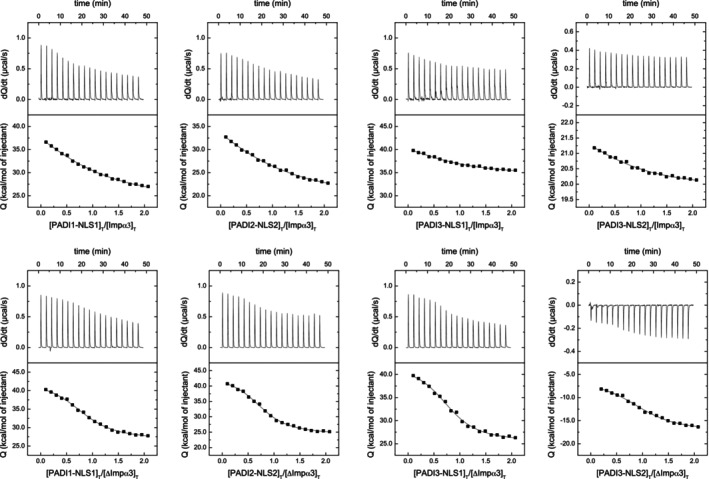
Quantitative determination of the binding of NLS peptides to either Impα3 or ΔImpα3 as monitored by ITC: Calorimetric titrations of Impα3 and ΔImpα3 with different NLS peptides. Upper panels show the thermograms (thermal power as a function of time) and lower panels show the binding isotherms (ligand‐normalized heat effects per injection as a function of the molar ratio in the calorimetric cell). Continuous curves correspond to the non‐linear least‐squares fittings according to a single ligand binding site interaction model. Experiments were carried out at 25°C.

The calorimetric titrations with the NLS peptides revealed that all of them were capable of binding to each importin species, Impα3 and ΔImpα3, with an unfavorable enthalpic contribution (Figure 6). Thus, the binding of the importins with the NLS regions was entropically driven. The values of the *K*_d_ were larger for the binding reaction with Impα3 (~10 μM) than for that with ΔImpα3 (~1 μM) (Table 3); thus, the NLS peptides had a higher affinity for ΔImpα3. This is due to the removal of the 60‐residue‐long IBB in the truncated protein species, which probably competes for the binding to the NLS‐recognition site present in importins, tending to hamper the association of the protein cargo through the NLS regions. The affinities determined by ITC were similar (i.e., within the same range) to those determined by fluorescence titrations in those cases where a reliable binding curve could be obtained (Table 2). The differences between the *K*_d_ values obtained by fluorescence and ITC can be rationalized as follows. In the fluorescence experiments, we pre‐mixed the solutions and we allowed them to come to equilibrium before the measurement. On the other hand, the ITC is a mixing experiment, and so it relies on a more reasonable fast kinetics during the binding reaction, or else the heat is released slowly and disappears into the background noise in the cell. Interestingly, the binding affinity of the two NLS regions previously predicted for PADI4 are similar to those determined for the NLSs derived from PADI1, PADI2, and PADI3, but the thermodynamic profile was the opposite: the interaction enthalpy was favorable and the entropic contribution was unfavorable for the case of PADI4, resulting in an enthalpically driven binding (Neira et al. 2022).

**TABLE 3:** Thermodynamic parameters of the binding of the importin species to the NLS peptides derived from PADI1, PADI2, and PADI3 as determined by ITC.

NLS peptides	Impα3				ΔImpα3			
	*K*_a_ (M^−1^)	*K*_d_ (μM)	Δ*H* (kcal mol^−1^)	*n*	*K*_a_ (M^−1^)	*K*_d_ (μM)	Δ*H* (kcal mol^−1^)	*n*
PADI1‐NLS1	1 × 10^5^	9.8	27.7	0.85	7.0 × 10^5^	1.4	15.9	0.87
PADI2‐NLS2	7.6 × 10^4^	13	34.4	0.96	8.7 × 10^4^	1.2	19.2	0.81
PADI3‐NLS1	1.6 × 10^5^	6.3	9.6	0.84	8.0 × 10^5^	1.3	16.7	0.82
PADI3‐NLS2	1.2 × 10^5^	8.1	2.8	0.85	6.0 × 10^5^	1.7	11.3	0.95

*Note*: Relative error in *K*_a_ and *K*_d_ is 30%, obtained from two measurements; the absolute error in Δ*H* is 0.4 kcal mol^−1^.

**FIGURE 7:**
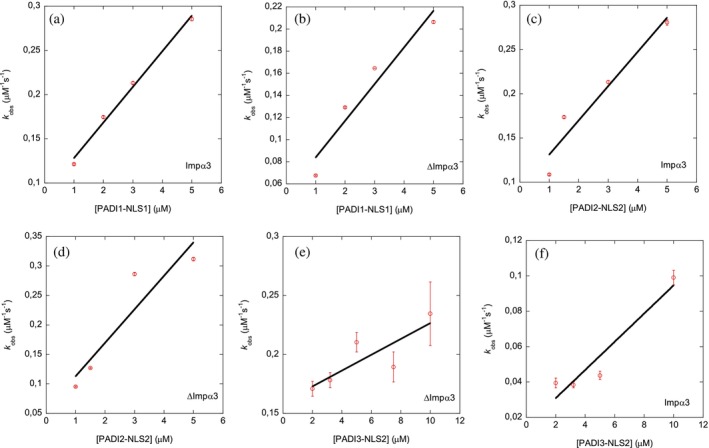
Binding of NLS peptides to either Impα3 or ΔImpα3 as monitored by BLI: Pseudo‐first order plot of the binding of all NLS peptides to each of the importin species, for those peptides where sensorgrams could be detected. The error bars of the fittings to Equation (5) at each peptide concentration are indicated. Experiments were carried out at 25°C.

Finally, we carried out BLI experiments to have another estimate of the affinity of the binding of the corresponding NLS peptide. With the help of this technique, we were able to determine an apparent affinity constant for each peptide. It is important to stress out that the apparent *K*_d_ (=*k*_off_/*k*_on_) values determined by using BLI data were obtained under the assumption that the binding of the peptide to each of the importin species could be modeled as a two‐state process (if there are one or more intermediate species in the binding reaction, then the *K*_d_ should be dependent on those other kinetic rates governing the in‐between reactions among those other species; Fersht 2017). Under this assumption, it could happen that some of the *K*_d_ values determined by BLI (Table 4) did not agree with those determined by fluorescence (Table 2) or ITC (Table 3). For PADI3‐NLS1, we could not observe reliable sensorgrams in the range of concentrations explored (1–5 μM). On the other hand, for PADI1‐NLS1, PADI2‐NLS2, and PADI3‐NLS2, we obtained reliable sensorgrams (Figures 7 and S3 and Table 4). For PADI1‐NLS1 and PADI2‐NLS2, the *k*_on_ and *k*_off_ values for the binding to Impα3 and ΔImpα3 were identical within the error, and therefore, the apparent affinity *K*_d_ values were the same within the error for both importin species. These affinity values determined by BLI were similar to those determined by ITC for ΔImpα3 (and therefore, smaller than those for Impα3). On the other hand, for the binding of PADI3‐NLS2 to Impα3, both the *k*_on_ and *k*_off_ rates were slower than those for PADI1‐NLS1 and PADI2‐NLS2; and for the binding to ΔImpα3, the *k*_on_ of PADI3‐NLS2 binding reaction was similar to that of the reaction with Impα3, but the *k*_off_ rate was one‐order of magnitude larger (0.16 s^−1^ vs. 0.014 s^−1^) (Table 4). The reason behind the lack of reliable sensorgrams for PADI3‐NLS1 (Table 4) was likely due to its smaller size (when compared to the other peptides); in fact, the MW of PADI3‐NLS1 is very close to the limit established by the BLI manufacturer to allow for detection: 3.0 kDa (Table 1).

**TABLE 4:** Kinetic parameters of the binding of importin species to the NLS peptides derived from PADI1, PADI2, and PADI3 determined by BLI.

NLS peptides	Impα3			ΔImpα3		
	*k*_on_ (μM^−1^ s^−1^)^a^	*k*_off_ (s^−1^)^a^	*K*_d_ (μΜ) (=*k*_off_/*k*_on_)^b^	*k*_on_ (μM^−1^ s^−1^)	*k*_off_ (s^−1^)	*K*_d_ (μΜ) (=*k*_off_/*k*_on_)^b^
PADI1‐NLS1	0.039 ± 0.003	0.09 ± 0.01	2.3 ± 0.5	0.03 ± 0.01	0.06 ± 0.03	2.0 ± 0.9
PADI2‐NLS2	0.038 ± 0.007	0.09 ± 0.02	2.4 ± 0.6	0.05 ± 0.01	0.06 ± 0.05	1.2 ± 0.7
PADI3‐NLS1	‐^c^	‐^c^	‐^c^	‐^c^	‐^c^	‐^c^
PADI3‐NLS2	0.008 ± 0.002	0.014 ± 0.010	2 ± 1	0.006 ± 0.002	0.16 ± 0.01	24 ± 12

^a^ Obtained from pseudo‐first order plots. Errors for *k*_on_ and *k*_off_ are fitting errors to Equation (6).

^b^ Errors were obtained from propagation errors for *k*_on_ and *k*_off_. The estimation of the *K*_d_ is carried out assuming that the binding was two‐state.

^c^ Binding was not observed during the association process in the sensorgrams.

### 2.3 NLS peptides targeted the NLS binding site in molecular simulations

One important point to clarify is whether our peptides mimicking the NLS of PADI proteins are actually anchoring to the canonical binding site of Impα3, just like the NLS of any other cargo protein. This is expected to be hard to investigate through standard structural techniques, such as crystallography, NMR and cryo‐electron‐microscopy, due to (i) the disordered nature of the NLS peptides evidenced by our experiments (section 2.1) and (ii) the range value of the affinity constants measured in the binding to Impα3. In addition, the use of NMR to elucidate the binding region in importin would implicate the full‐assignment of Impα3, which for a protein of that size is a formidable challenge. On the other hand, this question is difficult to elucidate as well in molecular simulations, because of the high conformational flexibility (≥100 rotational degrees of freedom) of the NLS peptides.

**FIGURE 8:**
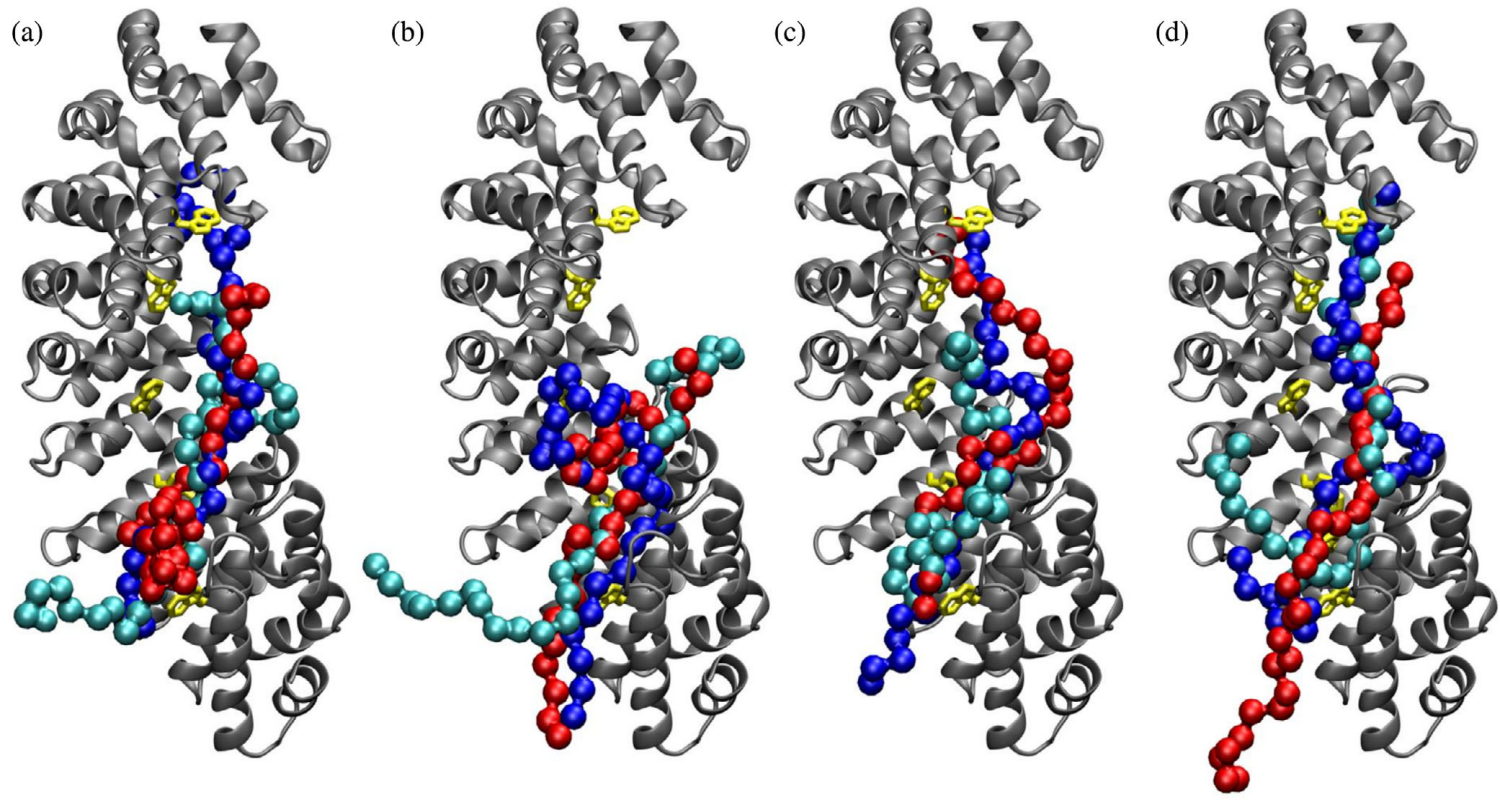
Binding of NLS peptides on Impα3 obtained in molecular docking simulations: Importin is shown in ribbon structure (gray), with tryptophan residues in stick representation (yellow). (a) PADI1‐NLS1 peptide; (b) PADI2‐NLS2 peptide; (c) PADI3‐NLS1 peptide; (d) PADI3‐NLS2 peptide. The first (blue), second (red), and third (cyan) most favorable docking poses are shown in all cases.

For tackling this challenging task, which is beyond the possibility of most traditional docking software engines (Eberhardt et al. 2021), we used the state‐of‐the‐art molecular docking algorithm DiffDock, a deep learning‐based diffusion generative model (Eberhardt et al. 2021). The structure of ΔImpα3 was used, without considering the IBB domain that would hamper the binding of the NLS peptide when self‐bound to the well‐folded importin domain, and it is otherwise little relevant when displaced by an NLS region. Figure 8 shows the results obtained by using DiffDock with default calculation parameters, for the molecular docking of the NLS peptides. In spite of the differences obtained among the various docking poses, it is clear that all of them target the internal curved region of Impα3, roughly along the sagittal plane of the protein structure. The confidence score (dimensionless and with arbitrary units) obtained for the most favorable poses was in between −3.5 and −4.0. These values are normally an indication of low confidence (<−1.5) for common binders, such as small drug‐like molecules with a single well‐defined binding mode, but are not surprisingly for our peptides, which are relatively long (each has 30 amino acid residues, except 25 residues for the sole PADI3‐NLS1) and with a disordered nature.

**FIGURE 9:**
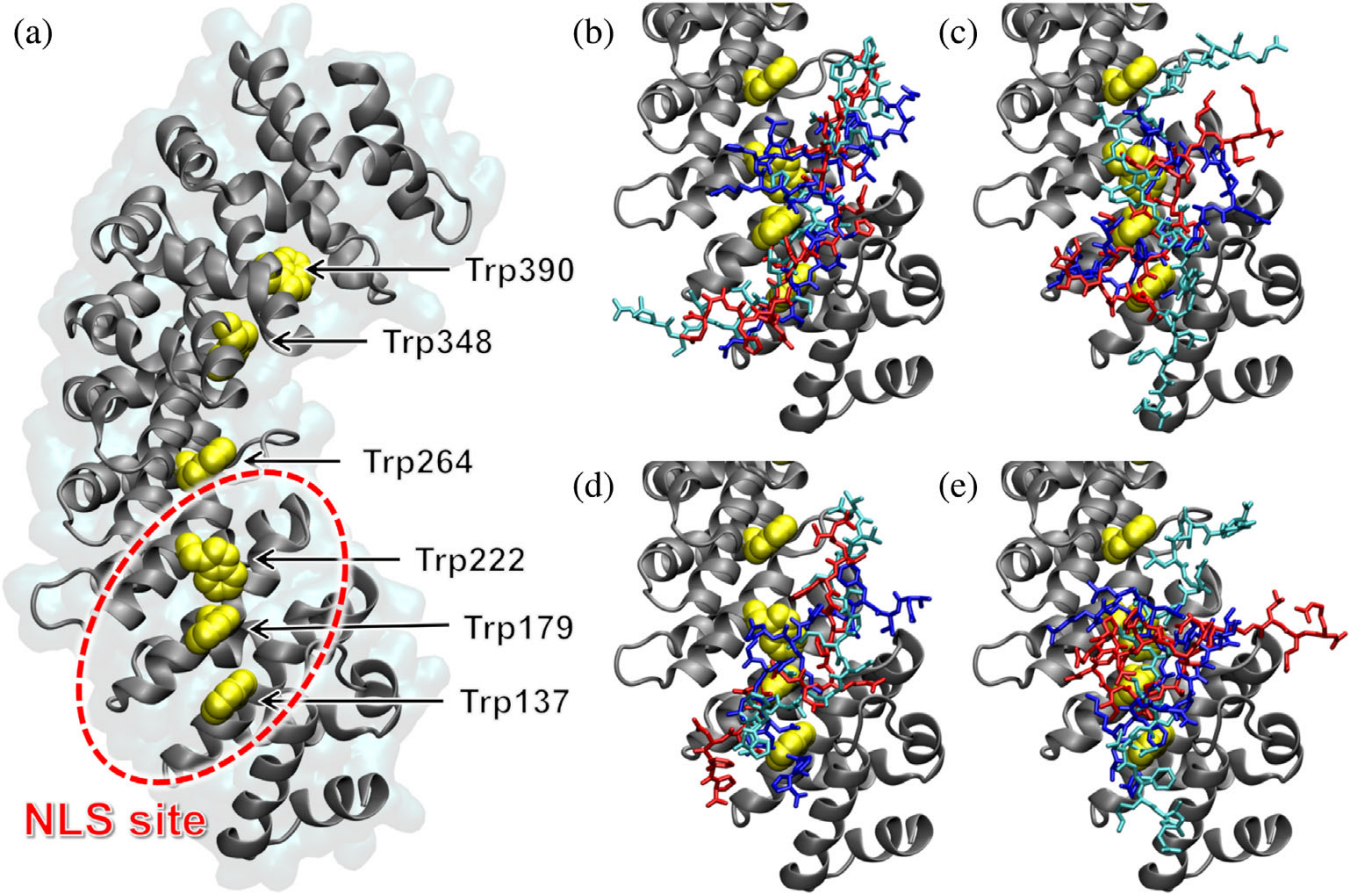
Binding of shortened NLS peptides on Impα3 obtained in molecular docking simulations: The peptides are shortened by five amino acids at both the N‐ and C‐terminal region. Impα3 is shown in ribbon structure (gray), with tryptophan residues in van der Waals representation (yellow). (a) Unliganded Impα3, with the canonical NLS binding site highlighted (red); (b) PADI1‐NLS1 peptide; (c) PADI2‐NLS2 peptide; (d) PADI3‐NLS1 peptide; and (e) PADI3‐NLS2 peptide. The first (blue), second (red), and third (cyan) most favorable docking poses are shown in all cases.

We also studied the binding to Impα3 of a truncated form of our peptides, with the length shortened by five amino acids both on the N‐terminal and C‐terminal regions, with a twofold aim: (i) to further reduce the computational complexity in the docking simulations and (ii) to determine more accurately whether the canonical NLS binding site of Impα3 is the hot‐spot targeted by the core region of the predicted NLS sequences. The results reported in Figure 9 show that the all four NLS peptides, even in the shortened version, created contacts with the tryptophan residues Trp137, Trp222, and Trp264, which are key amino acids that support the anchoring of NLSs to Impα3. The confidence scores obtained were in between −2.8 and −3.5, which were noticeably more favorable compared to the values found for the full‐length peptides, especially given the still large number of degrees of freedom even for the shortened peptides (i.e., 54–86 rotatable dihedral angles) and the completely blind (i.e., site‐agnostic) nature of the calculations performed.

To test the sensitivity of the simulation results with respect to the starting configuration of the shorter NLS peptides (including their initial position, distance from the target host, and initial conformation), we also performed docking runs by using the protein fragments extracted from the AlphaFold predictions of the PADI isoenzymes. The results reported in Figure S4 show that, even starting from the occluded‐state conformation of the NLS fragments in the native structure of the PADI isoforms, the docking poses of the peptides were mostly extended and largely in contact with the solvent. On the other hand, they all clearly targeted the tryptophan residues in the active site of Impα3. The confidence scores obtained were in between −2.6 and −3.4, which were also in the same range as those obtained starting from the short NLS peptides in elongated form.

In summary, the overall results of the docking simulations, in spite of the challenging nature of the binding process investigated, revealed that all the NLS peptides investigated target a single well‐defined hot‐spot on the surface of Impα3, which corresponded to the binding site expected for the NLS of cargo proteins. The binding involved a number of tryptophan residues on the surface of Impα3, as already suggested by our fluorescence titration experiments (section 2.2). These in silico results suggested that the four predicted NLSs had the binding features necessary to be tag sequences for the nuclear translocation of PADI1, PADI2, and PADI3. No attempt was made to more accurately estimate by computational methods the binding free energy and their individual contributions of each amino acid residues, given the disordered nature of the NLS peptides evident in our wet‐laboratory experiments, combined to the stochastic nature and inherent uncertainties in the diffusion procedure of the DiffDock algorithm (Eberhardt et al. 2021). Nevertheless, the comparison of the results obtained in simulation runs starting from different initial conditions (Figures 8, 9, and S4, being Figures 9 and S4 highly similar, even though the starting conditions were different) seems to suggest that the NLS peptides had a high degree of disorder and were mostly exposed to the solvent even in their importin‐bound state.

## 3 DISCUSSION

The five isoforms of the PADI family are enzymes that operate a post‐translational modification of arginine to citrulline. These proteins govern key cellular processes and are involved in numerous important diseases. So far, PADI4, as well as PADI2 under some conditions (Cherrington et al. 2010; Cherrington et al. 2012; Horibata et al. 2017; Zhang et al. 2012; Zheng et al. 2019), were the sole members of the PADI family experimentally observed both in the nucleus and cytoplasm. However, citrullination of histones by PADI1 is key to early embryo development (Zhang et al. 2016). Moreover, PADI3 intervenes in the direct regulation of enzymes involved in the transcription of genes related to cell migration, invasion, and survival, working as an oncogene and tumor suppressor (Chang et al. 2019; Mansouri et al. 2024; Uysal‐Onganer et al. 2021; Zhu et al. 2023). Therefore, judging from their functions and from their biological substrates, it seems that all these proteins should be translocated to the nucleus during the cell life time. It is important to indicate at this point that we have used as an in vitro nuclear translocation model Impα3, due to the reasons pinpointed above (section 1), but it does not mean that within the cell, any of the PADI isoforms could not be translocated by another nucleocytoplasmic transporter.

By using peptides synthesized on the basis of the NLS predictor cNLS Mapper, our in vitro biophysical results show (i) the putative NLS region of PADI2; and (ii) perhaps more interestingly, a putative NLS for PADI1 and two possible NLSs for PADI3. These four regions were capable of interacting with Impα3, a nuclear carrier of the importin family, and its IBB domain‐depleted species, ΔImpα3. The molecular docking simulations also demonstrated that the NLS peptides had a distinct ability to target the canonical binding site for the NLS of cargo proteins in the N‐terminal region of the folded domain of Impα3. Overall, all these findings provide a clear indication that the four predicted NLSs possessed the molecular features that are typical of tag signals for the nuclear translocation of the PADI protein species they belong to.

We measured the binding affinity of the NLS peptides for a specific importin, Impα3, for which we had already measured the affinity towards other cargos (Neira et al. 2020a; Neira et al. 2020b; Neira et al. 2021; Neira et al. 2022). However, we cannot rule out that the other predicted NLS regions in PADI1, PADI2, and PADI3, which were insoluble as isolated polypeptides, could also be real NLSs and then, any of the predicted regions (section 4.3) might be used during nuclear translocation, indistinctly, depending on the environmental conditions, and so PADI proteins can modulate their binding to importins under different circumstances.

It is interesting to compare the values of *k*_on_ and *k*_off_ rates obtained here (Table 4) with those measured for other isolated NLS peptides. For instance, in the canonical 31‐residue‐long NLS region of PADI4, for its binding to Impα3, the *k*_on_ was 0.0037 ± 0.0002 μM^−1^ s^−1^ and the *k*_off_ was 0.0685 ± 0.0007 s^−1^; whereas for the binding to ΔImpα3 the *k*_on_ was 0.011 ± 0.002 μM^−1^ s^−1^ and *k*_off_ was 0.046 ± 0.005 s^−1^ (Neira et al. 2022). These values were different from those measured in any of the peptides assayed in this work (Table 4), and only the *k*_on_ rates for both Impα3 and ΔImpα3 of PADI3‐NLS2 were similar to those of the NLSs from PADI4. Similarly, for the 32‐residue‐long non‐canonical NLS region of PADI4, in its binding to Impα3, the measured *k*_on_ was 0.003 ± 0.002 μM^−1^ s^−1^ and the *k*_off_ was 0.32 ± 0.01 s^−1^; whereas for the binding to ΔImpα3 the *k*_on_ was 0.020 ± 0.003 μM^−1^ s^−1^ and *k*_off_ was 0.23 ± 0.01 s^−1^ (Neira et al. 2022). These values, except the *k*_off_ rate of PADI3‐NLS2 when binding to ΔImpα3 (Table 4), were different from those measured in this work for any of the NLS regions. Thus, it is not possible to obtain a simple relationship among the kinetic rates, as measured by BLI, in the binding of the importin species to their different PADI cargoes.

We also demonstrated that the four isolated NLS peptides were disordered and they did not have any propensity to acquire helix‐ or turn‐like conformations (Table 1). Previous studies with other NLSs of intrinsically disordered proteins (Neira et al. 2020a; Neira et al. 2020b; Neira et al. 2021) or, alternatively, of folded proteins (Kobe 1999; Smith et al. 2018), have suggested an inhibitory action of the IBB: this domain hampers the anchoring of the NLS of the corresponding cargo protein into the major NLS‐binding region of Impα3. In this context, we have observed a stronger binding affinity of the four NLS regions for ΔImpα3 compared to Impα3 (Table 3), in agreement with the findings obtained for other NLS peptides assayed before (Neira et al. 2020a; Neira et al. 2020b; Neira et al. 2021). This difference between the values of the affinity constants for the two importin species is likely due to the fact that the 60‐residue‐long IBB is competing with the NLS for the NLS‐anchoring region. It is important to stress that to get these conclusions we have focused on the results obtained by ITC (Table 3), which is the gold standard in determining the thermodynamic parameters of a binding reaction. The differences in the *K*_d_ values from ITC with those obtained by fluorescence (Table 2) or by BLI (Table 4) have been explained before (section 2).

Our work also makes several predictions about the possible NLS regions present in PADI1, PADI2, and PADI3, although we cannot rule out that the insoluble peptides could also be the true NLSs in the corresponding intact protein. These predictions should be validated in future works by the use of protein engineering methods to elucidate the importance of particular PADI1, PADI2, or PADI3 residues on the binding to importin species. The PADI2‐NLS2 region could be responsible for the nuclear translocation of the intact PADI2, which leads to the citrullination of some histones (Neira et al. 2020a; Neira et al. 2020b; Neira et al. 2021; Neira et al. 2022) during the development of several types of cancers. The PADI1‐NLS1 region could be involved in the nuclear translocation of PADI1, leading to the citrullination of histones critical to early embryo development (Zhang et al. 2016). And finally, PADI3, for which a nuclear function has not been clearly established yet, could have PADI3‐NLS1 or PADI3‐NLS2 as two distinct NLS regions that might allow, under different cellular conditions, its nuclear translocation.

## 4 MATERIALS AND METHODS

### 4.1 Materials

Imidazole, Trizma base, DNase, SIGMAFAST protease inhibitor tablets, NaCl, Ni^2+^‐resin, 3‐(trimethylsilyl) propionic acid‐2,2,3,3‐^2^H_4_‐sodium salt (TSP) and ultra‐pure dioxane were from Sigma (Madrid, Spain). The β‐mercaptoethanol was from BioRad (Madrid, Spain). Ampicillin and isopropyl‐β‐D‐1‐thiogalactopyranoside were obtained from Apollo Scientific (Stockport, UK). Triton X‐100, dialysis tubing with a molecular weight cut‐off of 3500 Da, and the SDS protein marker (PAGEmark Tricolor) were from VWR (Barcelona, Spain). Amicon centrifugal devices with a molecular weight cut‐off of 30 kDa were from Millipore (Barcelona, Spain). The rest of materials were of analytical grade. Water was deionized and purified on a Millipore system.

### 4.2 Protein expression and purification

Impα3 and ΔImpα3 were purified as described before (Díaz‐García et al. 2020; Neira et al. 2020a; Neira et al. 2020b; Neira et al. 2021; Neira et al. 2022). Protein concentrations were determined by UV absorbance, employing an extinction coefficient at 280 nm estimated from the number of tyrosines and tryptophans in both importin species (six tyrosines and six tryptophans) (Gill and von Hippel 1989).

### 4.3 Prediction and synthesis of the putative NLS regions of PADI1, PADI2 and PADI3 proteins

The NLS regions for PADI1, PADI2, and PADI3 sequences were predicted by using the web server cNLS Mapper (Kosugi et al. 2009a; Kosugi et al. 2009b), available at http://nls-mapper.iab.keio.ac.jp (accessed on 10 June 2024). The results pointed out the existence of several possible NLS regions for each of the three isozymes (corresponding to prediction scores ≥3.5). As a guideline, larger scores are associated with a higher confidence in NLS prediction, with scores in the range 3–5 corresponding to proteins likely localized both in the nucleus and the cytoplasm, and scores above 7 corresponding to exclusive, or at least partial, nuclear localization (Kosugi et al. 2009a; Kosugi et al. 2009b). To allow for a comparison, the predicted regions in PADI4 (Neira et al. 2022), by using the same server, yielded a score of 5.3 for the canonical NLS and 6.1 for the non‐canonical one. The results for PADI1, PADI2, and PADI3 were as follows:

1. For PADI1, there were two predicted regions:

K^520^HQAKRSINEMLADRHLQRDNLHAQKCIDW^549^ (score 5.7).

R^551^NVLKRELGLAESDIVDIPQLFFLKNFYAE^580^ (score 5.3).

2 For PADI2, there were three predicted regions:

F^40^SLKHSEHVWVEVVRDGEAEEVATNGKQRW^69^ (score 3.6).

K^499^LFREKQKDGHGEAIMFKGLGGMSSKRITI^528^ (score 3.7).

S^523^KRITINKILSNESLVQENLYFQRCLDW^550^ (score 3.9).

3 Finally, for PADI3, there were two predicted regions:

P^360^HKTLPVVFDSPRNGELQDFPYKRILGP^387^ (score 5.3).

R^552^EVLKRELGLAECDIIDIPQLFKTERKKATA^582^ (score 6.3).

All these regions were synthesized as isolated acetylated and amidated peptides, to avoid possible structural fraying at both termini, with the following minor cost‐effective mutations designed to optimize solubility, oxidation propensity, and the quantitative determination of the amount of peptide used by employing UV absorption, as detailed hereafter.

In the first predicted NLS of PADI1, the wild‐type Cys546 was changed to Ser546 to avoid oxidation processes, leading to sequence K^520^HQAKRSINEMLADRHLQRDNLHAQKSIDW^549^. The peptide was soluble in aqueous solution at physiological pH (where the importin species are stable and acquire a native‐like structure; Díaz‐García et al. 2020). From now on, this peptide will be called PADI1‐NLS1.

The peptide corresponding to the second predicted NLS region of PADI1 had the unmodified sequence: R^551^NVLKRELGLAESDIVDIPQLFFLKNFYAE^580^. The peptide was not soluble in aqueous solution at physiological pH, and then it was not further considered. The same criterion was applied to the other insoluble peptides obtained (see below).

The peptide corresponding to the first predicted NLS region of PADI2 had the unmodified sequence: F^40^SLKHSEHVWVEVVRDGEAEEVATNGKQR^69^. When its solubility was assayed in aqueous solution at physiological pH, the peptide was not soluble.

In the second NLS of PADI2, a tyrosine was added at the end of the predicted sequence to allow for a more reliable spectrophotometric quantification of its concentration in solution (Gill and von Hippel 1989). This yielded the peptide sequence K^499^LFREKQKDGHGEAIMFKGLGGMSSKRIT^527^Y. The peptide was soluble at physiological pH in water. From now on, this peptide will be called PADI2‐NLS2.

In the third predicted NLS of PADI2, the wild‐type Cys547 was changed to Ser547 to avoid oxidation processes and, as indicated above, the C‐terminal Trp550 was removed to avoid solubility problems, obtaining the sequence S^523^KRITINKILSNESLVQENLYFQRSLD^549^. The peptide was not soluble in aqueous solution at physiological pH.

The peptide corresponding to the first predicted NLS region of PADI3 had the sequence: H^361^KTLPVVFDSPRNGELQDFPYKRIL^385^, where we removed Pro359, Gly386, and Pro387, as indicated above, to avoid solubility issues; the peptide was soluble at physiological pH. From now on, this peptide will be called PADI3‐NLS1.

In the second predicted NLS of PADI3, Cys564 was changed to Ser564 to avoid oxidation processes, and we removed Ala582 to avoid solubility problems, yielding the sequence R^552^EVLKRELGLAESDIIDIPQLFKTERKKAT^581^. In this peptide, we did not add a tyrosine at the C‐terminus, trying to avoid solubility problems. When assayed in aqueous solution at physiological pH, the peptide was soluble. From now on, this peptide will be called PADI3‐NLS2.

Therefore, we had four peptides, corresponding to potential NLS regions: PADI‐NLS1 (one out of two peptides from PADI1), PADI2‐NLS2 (one out of three peptides derived from PADI2), and the two peptides PADI3‐NLS1 and PADI3‐NLS2 (two out of two peptides derived from PADI3). The NLS regions within the whole structure of the three PADI proteins are shown in Figure S1; as it can be seen, all of them were disordered.

The peptides were produced by GenScript (Leiden, Netherlands), with a purity larger than 95%. Peptide concentrations were determined from the absorbance of tyrosines, tryptophans (measured at 280 nm) (Gill and von Hippel 1989), or phenylalanines (measured at 258 nm) (Pace and Schmid 1997) located in their sequences.

### 4.4 Fluorescence

#### 4.4.1 Steady‐state fluorescence

Fluorescence spectra were collected on a Cary Varian spectrofluorometer (Agilent, Santa Clara, CA), thermostatized with a Peltier unit.

In the steady‐state fluorescence experiments, the concentration of each peptide was 20 μM, and those of either importin species were 2 μM. Samples containing the isolated peptides, the isolated importin species, and the corresponding mixtures (at the concentrations indicated above) were prepared. Experiments were performed with samples in 50 mM Tris buffer, pH 8.0 at 25°C. Fluorescence experiments were repeated in triplicates with newly prepared samples. Variations of results among the experiments were lower than 5%. A 1‐cm‐pathlength quartz cell (Hellma, Kruibeke, Belgium) was used.

Experimental fluorescence parameters and the protocols used have been described elsewhere (Neira et al. 2016), with samples being excited at 280 or 295 nm. Appropriate blank corrections were made in all spectra.

#### 4.4.2 Titration experiments

For the titration of either Impα3 or ΔImpα3 with the NLS peptides, increasing concentrations of the latter, in the range 0–30 μM, were added to a solution with a fixed amount of either Impα3/ΔImpα3 (3 μM in protomer units). Experiments were carried out at pH 8.0 (50 mM, Tris buffer) at 25°C. The samples were excited at 280 and 295 nm, and the experimental setup was the same as in the steady‐state fluorescence experiments. In all cases, the appropriate blank corrections were subtracted. Spectra were corrected for inner‐filter effects during fluorescence excitation (Birdsall et al. 1983). Each titration (either Impα3 or ΔImpα3 with a peptide) was repeated three times, using newly prepared samples. In all cases, the variations in the results were lower than 10%.

The dissociation constant for each complex, *K*_d_, was calculated by fitting the binding isotherm constructed by plotting the observed fluorescence change as a function of peptide concentration to the general binding model, explicitly considering peptide depletion due to the binding (Beckett 2011; Royer and Scarlata 2008),

(1) $$F=\begin{array}{l} F_{0}+\frac{∆F_{\max}}{2{\left[\text{Impα}3_{\text{species}}\right]}_{T}}(({\left[\text{Impα}3_{\text{species}}\right]}_{T}\\ +\;{\text{[NLSpeptide]}}_{T}+K_{d})-\sqrt{\left(\begin{array}{c} {\left({\left[\text{Impα}3_{\text{species}}\right]}_{T}+{\text{[NLSpeptide]}}_{T}+K_{d}\right)}^{2}-\\ 4{\left[\text{Impα}3_{\text{species}}\right]}_{T}{\text{[NLSpeptide]}}_{T} \end{array}\right)}), \end{array}$$

where *F* is the measured fluorescence at any particular concentration of the corresponding peptide after subtraction of the corresponding blank; Δ*F*_max_ is the largest change in the fluorescence of the equivalent peptide when all polypeptide molecules were forming the complex, compared to the fluorescence of each isolated chain; *F*_0_ is the fluorescence intensity when no NLS peptide was added; [NLSpeptide]_T_ is the total concentration of the corresponding peptide, which was varied during the titration; and [Impα3_species_]_T_ is the constant concentration of either Impα3 or ΔImpα3. Fitting to the above equation was carried out by using KaleidaGraph (Synergy software, Reading, PA).

### 4.5 Far‐UV circular dichroism

Isothermal far‐UV circular dichroism (CD) spectra were collected on a Jasco J810 spectropolarimeter (Jasco, Tokyo, Japan) with a thermostated cell holder and interfaced with a Peltier unit at 25°C. The instrument was periodically calibrated with (+)‐10‐camphorsulfonic acid. A cell of pathlength 0.1 cm was used (Hellma, Kruibeke, Belgium). All spectra were corrected by subtracting the corresponding baseline. Concentration of each polypeptide and the buffers were the same used in the fluorescence experiments (section 4.4.1).

Spectra of each isolated macromolecule and those of their complexes with the corresponding peptides were acquired as an average of 6 scans, at a scan speed of 50 nm/min, with a response time of 2 s and a bandwidth of 1 nm. Sample handling and protocols have been described elsewhere (Neira et al. 2016).

### 4.6 Nuclear magnetic resonance

The nuclear magnetic resonance (NMR) spectra were acquired at 10°C on a Bruker Avance 500 MHz spectrometer (Bruker GmbH, Karlsruhe, Germany), equipped with a triple resonance probe and z‐pulse field gradients. Spectra were processed with Bruker TopSpin 2.1 (Bruker GmbH, Karlsruhe, Germany). All NMR experiments were carried out in 50 mM sodium phosphate buffer, at pH 7.2. Spectra were calibrated with TSP, by considering pH‐dependent changes of its chemical shifts (Cavanagh et al. 2007); probe temperature was calibrated with pure methanol (Cavanagh et al. 2007).

#### 4.6.1 1D‐ ^1^H‐NMR spectra

A number of 128 scans were acquired with 16 K acquisition points for the homonuclear 1D‐^1^H‐NMR spectra of each isolated peptide at ~1.2 mM concentration. The water signal was suppressed with the WATERGATE sequence (Piotto et al. 1992). The spectra were processed after zero‐filling (up to 32 K), apodized with an exponential window and base‐line corrected.

#### 4.6.2 Translational diffusion NMR (DOSY)

The peptide concentrations in DOSY experiments were ~100 μM, and 128 scans were acquired. Measurements of the translational self‐diffusion were performed with the pulsed‐gradient spin‐echo sequence in the presence of 100% D_2_O. Details on the experimental conditions, calibration of the gradient strength and fitting of the resulting curves have been described elsewhere (Neira et al. 2016). A final concentration of 1% of dioxane, which was assumed to have a hydrodynamic radius *R*_h_ of 2.12 Å (Wilkins et al. 1999), was added to the solution, as a reference to estimate the translational diffusion coefficient, *D*, of the peptides in solution.

#### 4.6.3 2D‐ ^1^H‐NMR spectra

Two‐dimensional spectra of the four peptides were acquired in each dimension in phase‐sensitive mode by using the time‐proportional phase incrementation technique (Marion and Wüthrich 1983) and a spectral width of 5500 Hz; peptide concentration was the same as used in the 1D‐^1^H‐NMR spectra. Standard TOCSY (with a mixing time of 80 ms) (Bax and Davis 1985) and NOESY experiments (with a mixing time of 250 ms) (Kumar et al. 1980) were performed by acquiring a data matrix size of 4096 × 512 points. The DIPSI (decoupling in the presence of scalar interactions) spin‐lock sequence (Cavanagh and Rance 1992) was used in the TOCSY experiments with a relaxation time of 1 s. A number of 96 scans were acquired per increment in the first dimension. NOESY spectra were collected with 128 scans per increment in the first dimension with a relaxation time of 1 s. In both spectra, the residual water signal was removed by using the WATERGATE sequence (Piotto et al. 1992). Data were zero‐filled, resolution‐enhanced with a square sine‐bell window function optimized in each spectrum, and baseline‐corrected. The ^1^H resonances were assigned by using the standard sequential assignment method (Wüthrich 1986). The chemical shift values of H_α_ protons in random‐coils were obtained taking into account neighboring residue effects (Kjaergaard et al. 2011; Kjaergaard and Poulsen 2011; Wüthrich 1986).

### 4.7 Isothermal titration calorimetry

The binding affinities for the NLS peptides with either Impα3 or ΔImpα3 were determined in a high‐sensitivity Auto‐iTC200 calorimeter (MicroCal, Malvern‐Panalytical, Malvern, UK). The titrations were carried out in phosphate buffer (pH 8.0, 50 mM). Protein solutions in the cell at 10 μM were titrated with the NLS solutions at 100 μM from the injection syringe. In each experiment a sequence of 19 injections of 2 μL of titrant solution spaced evenly over 150 s was programmed, with a stirring speed of 750 rpm and a reference power of 10 μcal/s.

Data analysis was performed by applying the single ligand binding site model (Vega et al. 2015). Briefly, the concentration of ligand and protein inside the calorimetric cell after each injection *j* were calculated as follows:

(2) $$\begin{array}{c} {\left[L\right]}_{T,j}={\left[L\right]}_{\mathrm{syr}}\left(1-\prod_{k=1}^{j}\left(1-\frac{v_{k}}{V_{0}}\right)\right)\\ {\left[P\right]}_{T,j}={\left[P\right]}_{\text{cell}}\prod_{k=1}^{j}\left(1-\frac{v_{k}}{V_{0}}\right), \end{array}$$

where [*L*]_syr_ is the concentration of ligand (peptide) in the syringe, [*P*]_cell_ is the initial concentration of protein in the cell, *v*_k_ is the volume of each injection, *V*_0_ is the cell volume, and *j* is the injection number. Normally, a factor *n* multiplying [*P*]_cell_ is included in Equation (2) to account for a fraction of non‐binding‐competent protein in the calorimetric cell. The concentration of complex *PL* after each injection was calculated by using the following expression:

(3) $$\left[\mathrm{PL}\right]=\frac{1+K_{a}{\left[P\right]}_{T}+K_{a}{\left[L\right]}_{T}-\sqrt{{\left(1+K_{a}{\left[P\right]}_{T}+K_{a}{\left[L\right]}_{T}\right)}^{2}-4K_{a}^{2}{\left[P\right]}_{T}{\left[L\right]}_{T}}}{2K_{a}}.$$

The binding isotherm (ligand‐normalized injection heats as a function of the molar ratio) was built by integrating the injection heat effects recorded in the thermogram (thermal power as a function of time), and the theoretical heat effect was calculated as follows:

(4) $$Q_{j}=\frac{1}{v_{j}{\left[L\right]}_{\mathrm{syr}}}V_{0}∆H\left({\left[\mathrm{PL}\right]}_{j}-{\left[\mathrm{PL}\right]}_{j-1}\left(1-\frac{v_{j}}{V_{0}}\right)\right)+Q_{d},$$

where [*PL*]_*j*_ is the concentration of complex formed after injection *j* (calculated with Equation (3)) and *Q*_*d*_ is the background injection heat (usually called “dilution heat,” but it includes many other unspecific phenomena such as mechanical mixing and buffer neutralization). The equilibrium association constant, *K*_a_ (and the dissociation constant, *K*_d_ = 1/*K*_a_), the binding enthalpy, Δ*H*, and the apparent binding stoichiometry, *n*, were estimated through nonlinear least squares regression analysis of the data performed in Origin 7.0 (OriginLab, Northampton, MA).

### 4.8 Biolayer interferometry

#### 4.8.1 Experimental design

The association (*k*_on_) and dissociation (*k*_off_) rate constants of the binding of the NLS peptides to either Impα3 or ΔImpα3 were determined by using a BLItz system (ForteBio, Pall, Barcelona, Spain) (Edwards et al. 1998; Frenzel and Willbold 2014; Hall et al. 1997; O'Shannessy and Winzor 1996; Pantoja‐Uceda et al. 2016). The buffer used in the experiments was that recommended by the manufacturer (Octet kinetics Buffer 10× (10× KB), PN 18‐1105, Sartorius). Since Impα3 and ΔImpα3 had a His‐tag, they were immobilized on His‐tag biosensors (Octet NTA Biosensors, PN 18‐5101) at 0.3 μM. Before the loading, each peptide was tested for binding at the largest concentration assayed against unloaded tips; in all the cases, we did not observe any binding. The peptide concentrations were in the range from 1 to 10 μM during the association step. The general scheme of the protein‐association/dissociation reactions in the BLItz system was 30 s of initial baseline acquisition with the 10× kinetics buffer; 120 s of loading the Impα3 or ΔImpα3 into the His‐tag biosensor; 30 s of baseline with the 10× kinetics buffer; 120 s of association of the corresponding peptide to the biosensor (which had been previously loaded with the corresponding importin species); and 120 s of dissociation of the corresponding NLS peptide from the biosensor‐immobilized‐Impα3/ΔImpα3. Baseline measurements of unloaded tips were subtracted from their matched measurement with the loaded tip.

#### 4.8.2 Fitting of the sensorgrams

Fitting of the sensorgrams was carried out as described elsewhere (Edwards et al. 1998; Frenzel and Willbold 2014; Hall et al. 1997; O'Shannessy and Winzor 1996; Pantoja‐Uceda et al. 2016). The experimentally interferometry response during the association step, *R*(*t*), after loading the biosensor with one of the importin species, under any condition was fitted as

(5) $$R\left(t\right)=R_{\mathrm{eq}}-R_{\mathrm{eq}}e^{\left(-k_{\mathrm{obs}}\;\left(t-t_{0}\right)\right)}-R{´}_{\mathrm{eq}}\left(t-t_{0}\right),$$

where the last term was included to consider the linear drift observed at longer times, where *R*_eq_ is the steady‐state (or equilibrium) response obtained at infinite time, when d*R*(*t*)/d*t* = 0, and *t*_0_ = 180 s is the time at which the association step started. The value of *k*_obs_ obtained at different peptide concentrations is given by the pseudo‐first order equation:

(6) $$k_{\mathrm{obs}}=k_{\mathrm{on}}\;\left[\text{NLSpeptide}\right]+k_{\mathrm{off}}.$$

where *k*_on_ and *k*_off_ are the association and dissociation rates, respectively, of the binding reaction of the corresponding NLS peptide to the importins.

The dissociation process was fitted with *R*(*t*) given by

(7) $$R\left(t\right)=R_{1}\;e^{\left(-k_{\mathrm{off}}\left(t-t_{0}\right)\right)}-R{\prime \prime }_{\mathrm{eq}}\left(t-t_{0}\right),$$

where *t*_0_ = 300 is the time at which the dissociation of the peptide from the biosensor‐bound Impα3/ΔImpα3 started in our experimental set‐up, *R*_1_ is the response level when dissociation starts and the last term, $R{\prime \prime }_{\mathrm{eq}}$, was included to consider the linear drift observed at longer times. Separated fittings of the association and the dissociation steps at the different peptide concentrations to Equations (5) and (7) were carried out by using KaleidaGraph. Fitting to linear Equation (6) (the so‐called pseudo‐first order plot) was carried out also with KaleidaGraph.

### 4.9 Molecular docking simulations

Molecular docking simulations were also performed to study the binding of the NLS peptides to Impα3. The protein was built as previously described (Hornos et al. 2025; Rizzuti et al. 2022) in the ΔImpα3 form, without the IBB domain that is a molecular competitor for the binding of NLS sequences. The template model was the crystallographic structure of Impα3 complexed with the NLS of the Ran‐binding protein 3, reported in the Protein Data Bank (PDB entry: 5XZX; Koyama and Matsuura 2017). The NLS peptides were built in elongated conformation (except for the turns in correspondence of proline residues) by using USCF Chimera, version 1.12 (Pettersen et al. 2004), including cappings to mimic the acetylated and amidated N and C terminus, respectively.

The algorithm DiffDock was used for performing molecular docking (Corso et al. 2022). This approach consists of a generative model on the manifold of docking poses of the peptides, combining a diffusion process to generate the ligand poses through roto‐translations and dihedral angle torsions, coupled with a confidence model to select the most favorable binding poses. A typical implementation was used, already applied to study the binding of peptides and other long‐chain molecules to PADI4 (Araujo‐Abad et al. 2025; Neira et al. 2025): 20 diffusion time divisions, 18 actual inference steps, and no final step noise applied. The resulting docking poses were displayed by using the molecular software VMD, version 1.9.3 (Humphrey et al. 1996).

To reduce the number of degrees of freedom in the peptides, besides simulations with the full‐length sequences (ranging from 25 to 30 amino acid residues), additional runs were performed with peptides shortened by five amino acids at both termini (from 15 to 20 residues), mimicking the innermost region of the predicted NLS sequences of PADI1, PADI2, and PADI3. Despite the reduced number of internal coordinates, the degrees of freedom in the shortened peptides (54–86 rotatable bonds) were still more than twice the number considered reliable for the use of other more traditional docking engines (Eberhardt et al. 2021), and reflected the large conformational flexibility expected for the peptides here studied. These simulation runs were performed both with the peptides in the elongated conformation built by using USCF Chimera and, alternatively, the conformation extracted from the structure of the three PADI isozymes reported in the AlphaFold database (Jumper et al. 2021). To accurately mimic the sequence of our shortened NLS peptide, the in silico mutation Cys564Ser was necessary for the sole PADI3‐NLS2 region; in fact, all the other mutations in the full‐length peptides were at either of their five‐residue termini, and did not affect the sequence of the shortened peptides.

## AUTHOR CONTRIBUTIONS

**José L. Neira:** Conceptualization; investigation; funding acquisition; writing – original draft; methodology; formal analysis; writing – review and editing; project administration. **Olga Abian:** Investigation; funding acquisition; writing – original draft; writing – review and editing; project administration; formal analysis. **Adrián Velazquez‐Campoy:** Investigation; funding acquisition; writing – original draft; writing – review and editing; formal analysis; project administration. **Bruno Rizzuti:** Investigation; funding acquisition; writing – original draft; writing – review and editing; formal analysis; project administration.

## CONFLICT OF INTEREST STATEMENT

The authors declare no conflicts of interest.

## Supporting information


**Figure S1.** Location of the predicted NLS region in the monomeric structure of PADI isoenzymes. (a) PADI1, NLS region 520–549 (orange), and 551–580 (red). (b) PADI2, NLS region 40–69 (purple), 499–528 (yellow), and 523–550 (orange). (c) PADI3, NLS region 360–387 (blue), and 552–582 (red). In the three PADI isoforms all these regions are labeled, as well as the backbone N and C terminus, and the catalytic cysteine residue in the center of the protein active site.
**Figure S2.** Conformational characterization of the NLS regions by 1D‐^1^H‐NMR spectra. The amide region of the spectra of the four isolated NLS regions. The signal appearing at ~8.00 ppm in some of the spectra of the peptides is an impurity from the synthesis.
**Figure S3.** Binding of selected NLS regions to importin species as measured by BLI. The association steps of different sensorgrams for each of the two importin species with several NLS peptides are shown. Experiments were carried out at 25°C.
**Figure S4.** Binding of short NLS peptides on Impα3 obtained in molecular docking simulations starting from fragments of PADI isoforms. The peptides are shortened by five amino acids at both the N‐ and C‐terminal region compared to the full‐length NLS peptides. Impα3 is shown in ribbon structure (gray), with tryptophan residues in van der Waals representation (yellow). (a) Unliganded Impα3, with the canonical NLS binding site highlighted (red); (b) PADI1‐NLS1 peptide; (c) PADI2‐NLS2 peptide; (d) PADI3‐NLS1 peptide; and (e) PADI3‐NLS2 peptide. The first (blue), second (red), and third (cyan) most favorable docking poses are shown in all cases.
**Table S1.** Chemical shifts (δ, ppm from TSP) of PADI1‐NLS1 in aqueous solution (pH 7.2, 10°C).
**Table S2.** Chemical shifts (δ, ppm from TSP) of PADI2‐NLS2 in aqueous solution (pH 7.2, 10°C).
**Table S3.** Chemical shifts (δ, ppm from TSP) of PADI3‐NLS1 in aqueous solution (pH 7.2, 10°C).
**Table S4.** Chemical shifts (δ, ppm from TSP) of PADI3‐NLS2 in aqueous solution (pH 7.2, 10°C).

## Data Availability

The data that support the findings of this study are available from the corresponding author upon reasonable request.
