# Supplementary material for: Unveiling nuclear localization signals in human arginine deiminase proteins
Source: Protein Sci. 2026 Mar 2;35(4):e70517. doi: 10.1002/pro.70517 (PMC12953195; doi:10.1002/pro.70517)
Supplement: Supplementary file 1 — Figure S1. Location of the predicted NLS region in the monomeric structure of PADI isoenzymes. (a) PADI1, NLS region 520–549 (orange), and 551–580 (red). (b) PADI2, NLS region 40–69 (purple), 499–528 (yellow), and 523–550 (orange). (c) PADI3, NLS region 360–387 (blue), and 552–582 (red). In the three PADI isoforms all these regions are labeled, as well as the backbone N and C terminus, and the catalytic cysteine residue in the center of the protein active site. Figure S2. Conformational characterization of the NLS regions by 1D‐1H‐NMR spectra. The amide region of the spectra of the four isolated NLS regions. The signal appearing at ~8.00 ppm in some of the spectra of the peptides is an impurity from the synthesis. Figure S3. Binding of selected NLS regions to importin species as measured by BLI. The association steps of different sensorgrams for each of the two importin species with several NLS peptides are shown. Experiments were carried out at 25°C. Figure S4. Binding of short NLS peptides on Impα3 obtained in molecular docking simulations starting from fragments of PADI isoforms. The peptides are shortened by five amino acids at both the N‐ and C‐terminal region compared to the full‐length NLS peptides. Impα3 is shown in ribbon structure (gray), with tryptophan residues in van der Waals representation (yellow). (a) Unliganded Impα3, with the canonical NLS binding site highlighted (red); (b) PADI1‐NLS1 peptide; (c) PADI2‐NLS2 peptide; (d) PADI3‐NLS1 peptide; and (e) PADI3‐NLS2 peptide. The first (blue), second (red), and third (cyan) most favorable docking poses are shown in all cases. Table S1. Chemical shifts (δ, ppm from TSP) of PADI1‐NLS1 in aqueous solution (pH 7.2, 10°C). Table S2. Chemical shifts (δ, ppm from TSP) of PADI2‐NLS2 in aqueous solution (pH 7.2, 10°C). Table S3. Chemical shifts (δ, ppm from TSP) of PADI3‐NLS1 in aqueous solution (pH 7.2, 10°C). Table S4. Chemical shifts (δ, ppm from TSP) of PADI3‐NLS2 in aqueous solution (pH 7.2, 10°C). [file PRO-35-e70517-s001.docx]

**Supplementary Material**

Unveiling nuclear localization signals in human arginine deiminase proteins

José L. Neira, Olga Abian, Adrián Velazquez-Campoy and Bruno Rizzuti

Figure S1: **Location of the predicted NLS region in the monomeric structure of PADI isoenzymes.** (A) PADI1, NLS region 520−549 (orange), and 551−580 (red). (B) PADI2, NLS region 40−69 (purple), 499−528 (yellow), and 523−550 (orange). (C) PADI3, NLS region 360−387 (blue), and 552−582 (red). In the three PADI isoforms all these regions are labelled, as well as the backbone N and C terminus, and the catalytic cysteine residue in the centre of the protein active site.


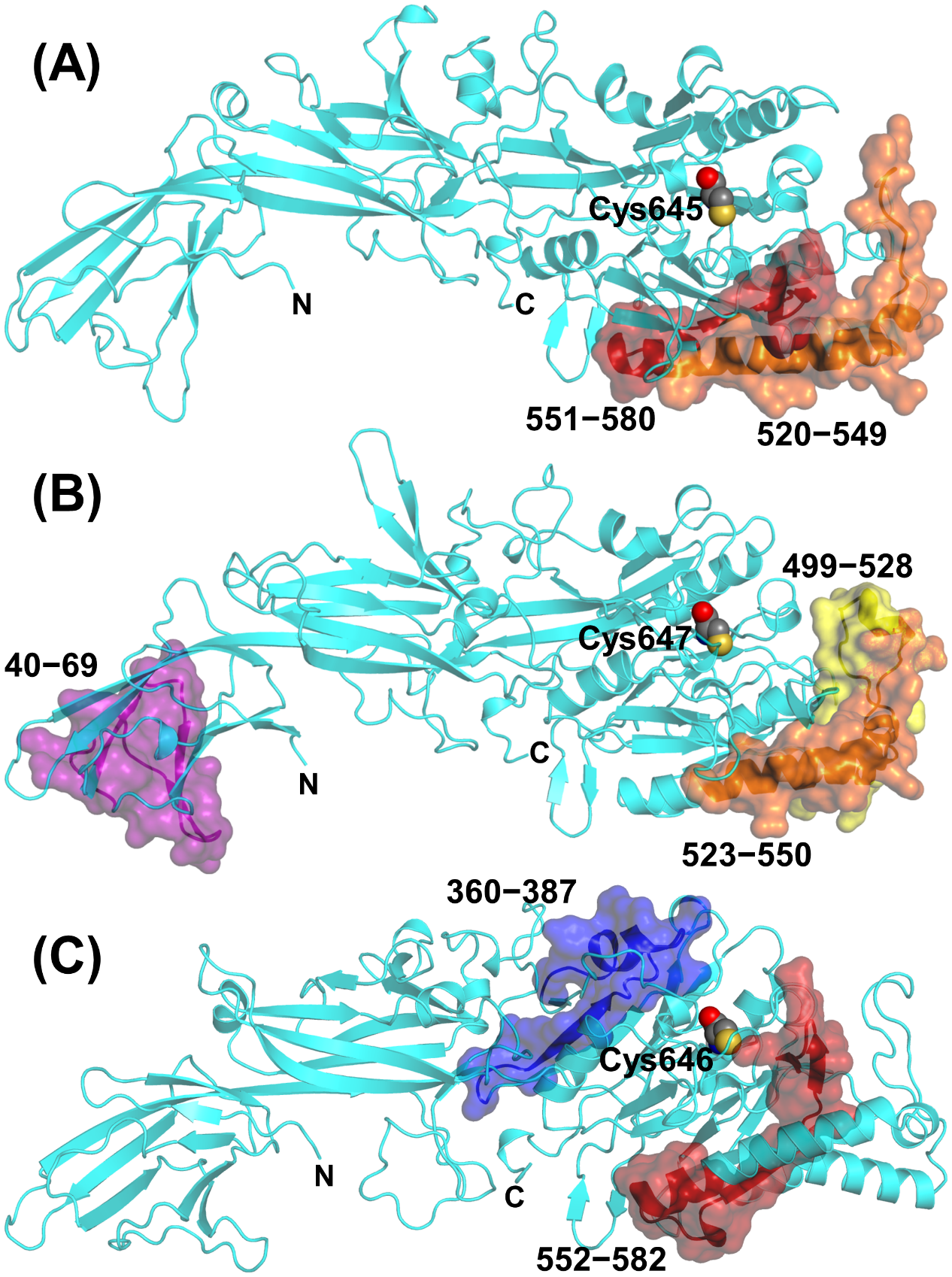


Figure S2: **Conformational characterization of the NLS regions by 1D-^1^H-NMR spectra.** The amide region of the spectra of the four isolated NLS regions. The signal appearing at ~8.00 ppm in some of the spectra of the peptides is an impurity from the synthesis.


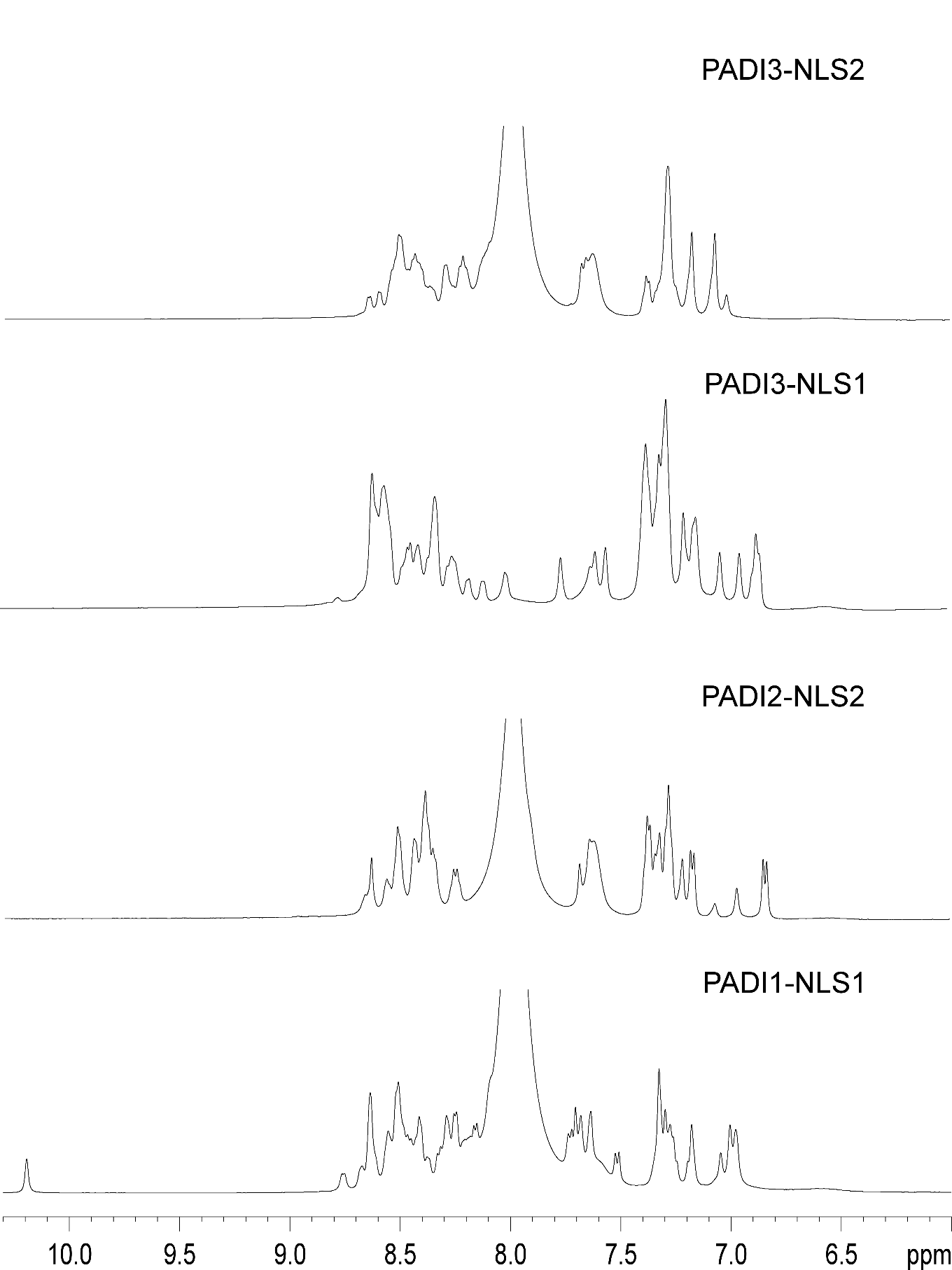


Figure S3: **Binding of selected NLS regions to importin species as measured by BLI**. The association steps of different sensorgrams for each of the two importin species with several NLS peptides are shown. Experiments were carried out at 25 ºC.

FIGURE S4: **Binding of short NLS peptides on Impα3 obtained in molecular docking simulations starting from fragments of PADI isoforms.** The peptides are shortened by five amino acids at both and N- and C-terminal region compared to the full-lenght NLS peptides. Impα3 is shown in ribbon structure (gray), with tryptophan residues in van der Waals representation (yellow). (A) Unliganded Impα3, with the canonical NLS binding site highlighted (red); (B) PADI1-NLS1 peptide; (C) PADI2-NLS2 peptide; (D) PADI3-NLS1 peptide; and (E) PADI3-NLS2 peptide. The first (blue), second (red), and third (cyan) most favorable docking poses are shown in all cases.


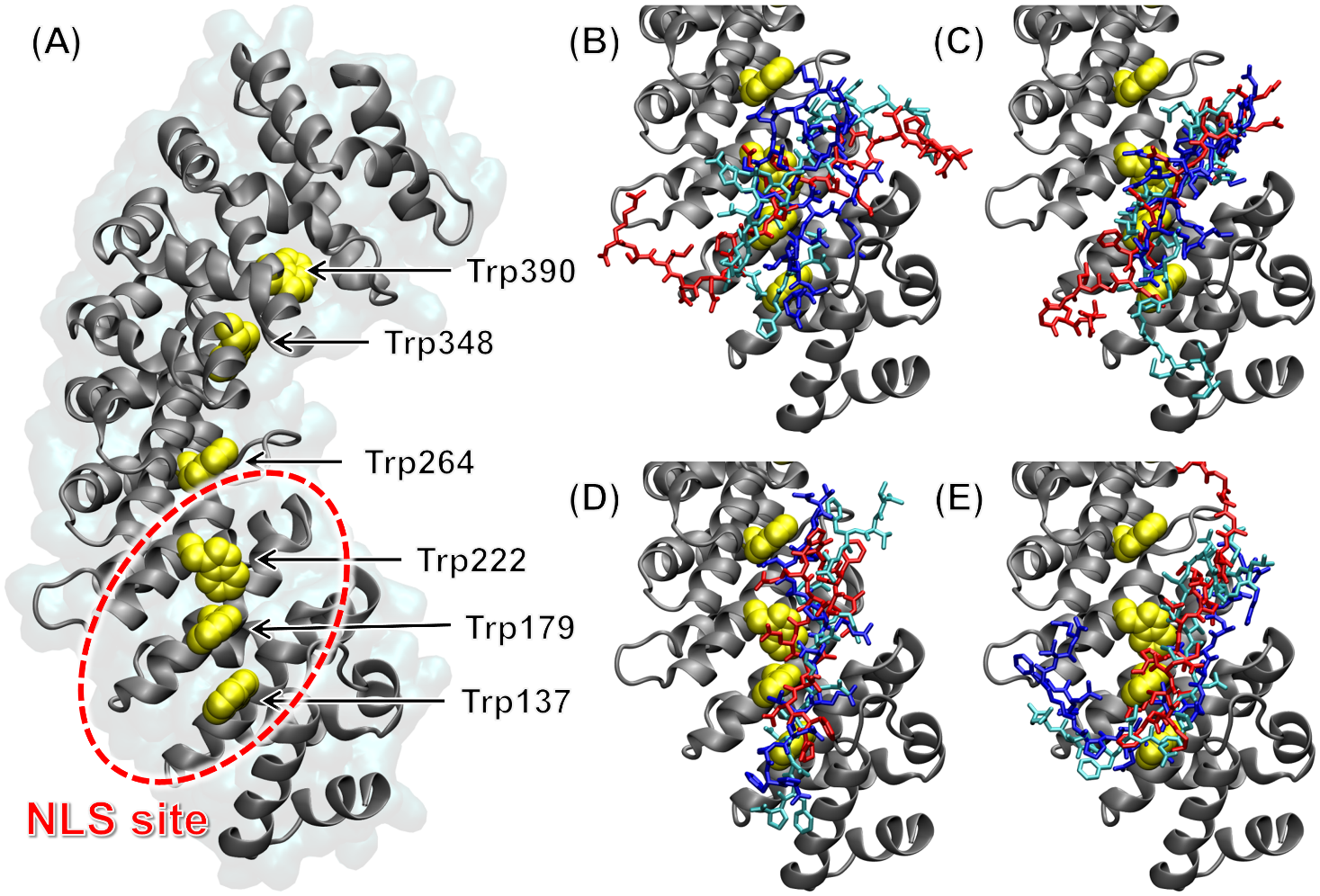


**SUPPPORTING TABLES**

| Table ST1: Chemical shifts (δ, ppm from TSP) of PADI1-NLS1 in aqueous solution (pH 7.2, 10 ºC)^a^ | | | | | | | | | | | | | | | |
| --- | --- | --- | --- | --- | --- | --- | --- | --- | --- | --- | --- | --- | --- | --- | --- |
|  | NH | H_α_ | H_β2_ | H_β3_ | H_γ2_ | H_γ3_ | H_δ2_ | H_δ3_ | | H_ε_ | | | | H_ζ_ | |
| Ac-Lys520 |  | 4.20 (-0.06) |  | |  | |  | | |  | | | |  | |
| His521 | 8.76 | 4.74 (0.10) | 3.24; 3.28 | |  | | 8.66 (C2H); 7.33 (C4H) | | | | | | | | |
| Gln522 | 8.51 | 4.32 (0.02) | 2.00 | | 2.38 | |  | | |  | | | |  | |
| Ala523 | 8.62 | 4.31 (0.04) | 1.43 (Me) | |  | |  | | |  | | | |  | |
| Lys524 |  | 4.20* |  | |  | |  | | |  |  | | |  | |
| Arg525 |  | 4.38 (0.00) | 1.82; 2.07 | | 1.72 | | 3.21 | | | 7.29 | | | |  | |
| Ser526 | 8.67 | 4.52 (-0.05) | 3.99 | |  | |  |  | |  |  | | |  | |
| Ile527 | 8.50 | 4.14 (-0.05) | 1.90; 0.93 (Me) | | 1.25; 1.49 | | 0.93 (Me) | | |  |  | | |  | |
| Asn528 | 8.50 | 4.66 (-0.03) | 2.79 | |  | | 7.05; 7.73 | |  | | | |  | | |
| Glu529 | 8.29 | 4.22 (-0.02) | 2.09 | | 2.36 | |  | | |  | | | |  | |
| Met530 | 8.37 | 4.39 (-0.09) | 2.15 | | 2.53; 2.67 | |  | | |  | | | |  | |
| Leu531 | 8.19 | 4.27 (-0.08) | 2.10 | | 1.74 | | 0.89 (Me) | | |  | | | |  | |
| Ala532 | 8.09 | 4.23 (-0.06) | 1.40 (Me) | |  | | | | | | | | | | |
| Asp533 | 8.22 | 4.58 (0.03) | 2.77 | |  | |  | |  | | | | | | |
| Arg534 | 8.25 | 4.33 (0.07) | 1.91 | | 1.69 | | 3.21 | | | 7.34 | | | |  | |
| His535 | 8.16 | 4.64 (0.00) | 3.26; 3.39 | |  | | 8.66 (C2H); 7.33 (C4H) | | | | | | | | |
| Leu536 | 8.24 | 4.28 (-0.04) | 1.66 | | 1.58 | | 0.83 (Me) | |  | | |  | | | |
| Gln537 | 8.44 | 4.30 (-0.04) | 2.03 | | 2.44 | |  | |  | | | | | | |
| Arg538 | 8.54 | 4.38 (0.06) | 1.87 | | 1.69 | | 3.21 | | 7.34 | | | | | |  |
| Asp539 | 8.52 | 4.67 (0.09) | 2.79 | |  | |  | |  | | | | | | |
| Asn540 | 8.46 | 4.65 (0.00) | 2.80 | |  | |  | | |  | | | |  | |
| Leu541 | 8.28 | 4.25 (-0.03) | 1.63 | | 1.63 | | 0.88 (Me) | | |  | | | |  | |
| His542 | 8.43 | 4.69 (0.05) | 3.24; 3.34 | |  | | 8.66 (C2H); 7.33 (C4H) | | | | | | | | |
| Ala543 | 8.29 | 4.26 (-0.03) | 1.41 (Me) | |  | |  | | |  | | | |  | |
| Gln544 | 8.57 | 4.31 (0.01) | 2.00 | | 2.38 | |  | | |  | | | |  | |
| Lys545 | 8.25* | 4.34* |  | |  | |  | | |  | | | |  | |
| Ser546 | 8.41 | 4.43 (-0.05) | 3.82 | |  | |  | | |  | | | |  | |
| Ile547 | 8.25 | 4.04 (-0.06) | 1.90; 0.83 (Me) | | 1.08; 1.36 | | 0.59 (Me) | | |  | | | |  | |
| Asp548 | 8.32 | 4.64 (0.11) | 2.59; 2.69 | |  | |  | | |  | | | |  | |
| Trp549-Am | 8.57 | 4.65 (0.00) | 3.30 | |  | | 10.19 (NH); 7.31 (C2H); 7.76 (C4H); 7.19 (C5H); 7.27 (C6H); 7.54 (C7H) | | | | | | | | |

^a^The (*) indicates those resonances which could not be unambiguously assigned. For the H_α_ proton column, the values within parenthesis are the conformational shifts (δ_res_-δ_rc_), only shown for those protons unambiguously assigned. For those resonances, which were not unambigously assigned, the conformational shifts were not calculated. The random-coil values for the sequence were obtained from: <https://spin.niddk.nih.gov/bax/nmrserver/Poulsen_rc_CS/>).

| Table ST2: Chemical shifts (δ, ppm from TSP) of PADI2-NLS2 in aqueous solution (pH 7.2, 10 ºC)^a^ | | | | | | | | | | | | | | | | |
| --- | --- | --- | --- | --- | --- | --- | --- | --- | --- | --- | --- | --- | --- | --- | --- | --- |
|  | NH | H_α_ | H_β2_ | H_β3_ | H_γ2_ | H_γ3_ | H_δ2_ | H_δ3_ | | | H_ε_ | | | | H_ζ_ | |
| Ac-Lys499 |  | 4.30 (0.07) | 1.75 | |  | |  | | | |  | | | |  | |
| Leu500 | 8.38 | 4.16 (-0.11) | 1.77 | | 1.68 | | 0.90 (Me) | | | |  | | | |  | |
| Phe501 | 8.34 | 4.60 (-0.06) | 3.09 | |  | | 7.28 | | | |  | | | |  | |
| Arg502 | 8.26 | 4.32 (0.03) | 1.80 | | 1.62 | | 3.19 | | | | 7.24 | | | |  | |
| Glu503 | 8.43* | 4.26* | 1.96* | | 2.39* | |  | | | |  | |  | |  | |
| Lys504 |  |  |  | |  | |  | | | |  | | | |  | |
| Gln505 | 8.52* | 4.31* | 2.05 | | 2.41 | |  |  | | | 6.97; 7.62 | | | |  | |
| Lys506 | 8.56 | 4.30 (0.00) | 1.79 | | 1.40 | |  | | |  | | | | | |  |
| Asp507 | 8.52 | 4.63 (0.05) | 2.75 | |  | |  | | | |  | | | |  | |
| Gly508 | 8.49 | 3.93 (-0.01) |  | |  | |  | | | |  | |  | |  | |
| His509 | 8.56 | 4.74 (0.07) | 3.29; 3.37 | |  | | 8.63 (C2H); 7.32 (C4H) | | | |  | | | |  | |
| Gly510 | 8.63 | 3.98 (-0.01) |  | |  | |  | | |  | | | | | |  |
| Glu511 | 8.39* | 4.34* | 2.04 | | 2.60* | |  | |  | | | | |  | | |
| Ala512 | 8.51 | 4.29 (-0.03) | 1.39 (Me) | |  | |  | | | |  | | | |  | |
| Ile513 | 8.23 | 4.11 (-0.06) | 1.82; 0.84 (Me) | | 1.13; 1.50 | | 0.84 (Me) | | |  | |  | | | |  |
| Met514 | 8.40 | 4.34 (0.09) | 2.04 | | 2.60 | |  | | |  | |  | | | |  |
| Phe515 | 8.35 | 4.63 (-0.03) | 3.07 | |  | | 7.28 | | |  | | | | | | |
| Lys516 |  |  |  | |  | |  | | |  | | | | | | |
| Gly517 | 8.44* | 3.90* |  | |  | |  | | |  | | | | | | |
| Leu518 | 8.38 | 4.38 (-0.01) | 1.74 | | 1.41 | | 0.77 (Me) | | | |  | | | |  | |
| Gly519 | 8.66 | 3.99(-0.1) |  | |  | |  | | | |  | | | |  | |
| Gly520 | 8.38 | 3.95 (-0.05) |  | |  | |  | | | |  | | | |  | |
| Met521 | 8.37* | 4.40* | 2.90* | |  | |  | | | |  | |  | |  | |
| Ser522 |  |  |  | |  | |  | | | |  | | | |  | |
| Ser523 |  |  | 3.96 | |  | |  | | | |  | | | |  | |
| Lys524 | 8.35 | 4.35 (0.03) | 1.71 | |  | |  | | | |  | | | |  | |
| Arg525 | 8.40 | 4.33 (0.02) | 1.80 | | 1.62 | | 3.19 | | | | 7.27 | | | |  | |
| Ile526 |  |  |  | |  | |  | | | |  | |  | |  | |
| Thr527 | 8.25 | 4.37 (0.06) | 4.17 | | 1.17 (Me) | |  | | | |  | |  | |  | |
| Tyr528-Am | 8.38 | 4.60 (-0.01) | 3.09 | |  | | 7.15 | | | | 6.86 | | | |  | |

^a^ The (*) indicates those resonances which could not be unambiguously assigned. The Tyr528 does not belong to the wild-type sequence of the protein. For the H_α_ proton column, the values within parenthesis are the conformational shifts (δ_res_-δ_rc_), only shown for those protons unambiguously assigned. For those resonances, which were not unambigously assigned, the conformational shifts were not calculated. The random-coil values for the sequence were obtained from: <https://spin.niddk.nih.gov/bax/nmrserver/Poulsen_rc_CS/>).

| Table ST3: Chemical shifts (δ, ppm from TSP) of PADI3-NLS1 in aqueous solution (pH 7.2, 10 ºC)^a^ | | | | | | | | | | | | | | | |
| --- | --- | --- | --- | --- | --- | --- | --- | --- | --- | --- | --- | --- | --- | --- | --- |
|  | NH | H_α_ | H_β2_ | H_β3_ | H_γ2_ | H_γ3_ | H_δ2_ | H_δ3_ | | H_ε_ | | | | H_ζ_ | |
| Ac-His361 |  | 4.60 (-0.05) | 3.08 | |  | | 8.56 (C2H); 7.18 (C4H) | | |  | | | |  | |
| Lys362 | 8.76 | 4.33 (-0.05) | 1.85 | | 1.46 | |  | | | 2.80 | | | |  | |
| Thr363 | 8.45 | 4.37 (0.01) | 4.18 | | 1.21 (Me) | |  | | |  | | | |  | |
| Leu364 | 8.53 | 4.67 (0.00) | 1.64 | | 1.64 | | 0.88 | | |  | | | |  | |
| Pro365 |  | 4.48 (0.00) | 2.05 | | 2.34 | | 3.76; 3.86 | | |  | |  | |  | |
| Val366 | 8.32 | 3.98 (-0.05) | 2.02 | | 0.80; 0.98 (Me) | |  |  | |  | |  | |  | |
| Val367 | 8.26 | 4.08 (0.02) | 2.05 | | 0.89 (Me) | |  |  | |  | |  | |  | |
| Phe368 | 8.54 | 4.68 (0.02) | 2.94; 3.16 | |  | | 7.28 | |  | | | | | |  |
| Asp369 | 8.48 | 4.66 (0.04) | 2.58 | |  | |  | | |  | | | |  | |
| Ser370 | 8.23 | 4.60 (-0.06) | 3.82 | |  | |  |  | |  | |  | |  | |
| Pro371 |  | 4.47 (0.01) | 2.03 | | 2.28 | | 3.69; 3.91 | | |  | | | |  | |
| Arg372 | 8.57* | 4.35* | 1.79 | | 1.65 | | 3.17 | | 7.28 | | | | | |  |
| Asn373 | 8.59 | 4.72 (-0.01) | 2.85 | | 7.02; 7.75 | | | | | | | | | | |
| Gly374 | 8.57 | 3.90 (-0.06) |  | |  | |  | | |  | | | |  | |
| Glu375 | 8.32 | 4.29 (0.01) | 1.95 | | 2.27 | |  | |  | |  | |  | | |
| Leu376 | 8.40 | 4.34 (-0.01) | 1.64 | | 1.64 | | 0.88 (Me) | |  | |  | |  | | |
| Gln377 | 8.41 | 4.29 (-0.07) | 1.95 | | 2.27 | | 6.92; 7.61 | |  | | | | | | |
| Asp378(t) | 8.33 | 4.59 (0.05) | 2.53 | |  | |  | |  | | | | | | |
| Asp378(c) | 8.37 | 4.51 | 2.60; 2.74 | |  | |  | |  | | | | | | |
| Phe379(t) | 8.17 | 4.80 (-0.08) | 2.89; 2.99 | |  | | 7.27 | | |  | | | |  | |
| Phe379(c) | 8.22 | 4.80 | 2.95; 3.41 | |  | |  | | |  | | | |  | |
| Pro380 |  | 4.39 (0.07) | 1.81; 2.09 | | 2.19 | | 3.44; 3.70 | | |  | |  | |  | |
| Tyr381 | 8.00 | 4.54 (0.05) | 3.03 | |  | | 7.14 | | | 6.88 | | | |  | |
| Lys382 | 8.10 | 4.28 (0.02) | 1.72 | | 1.40 | | 1.70 | | | 3.01 | | | | 7.64 | |
| Arg383 | 8.33* | 4.32 (0.05) | 1.81 | | 1.65 | | 3.17 | | | 7.32 | | | |  | |
| Ile384 | 8.35 | 4.13 (-0.02) | 1.86 | | 1.18; 1.56 (0.88 (Me)) | | 0.88 (Me) | | |  | |  | |  | |
| Leu-Am |  | 4.42 (0.07) | 1.71 | | 1.70 | | 0.93 (Me) | | |  | |  | |  | |

^a^ The (*) indicates those resonances which could not be unambiguously assigned. The (c) and (t) indicate the cis and trans conformations, respectively.The N terminus was acetylated and the C terminus was amidated. For the H_α_ proton column, the values within parenthesis are the conformational shifts (δ_res_-δ_rc_), only shown for those protons unambiguously assigned. For those resonances, which were not unambigously assigned, the conformational shifts were not calculated. The random-coil values for the sequence were obtained from: <https://spin.niddk.nih.gov/bax/nmrserver/Poulsen_rc_CS/>).

| Table ST4: Chemical shifts (δ, ppm from TSP) of PADI3-NLS2 in aqueous solution (pH 7.2, 10 ºC)^a^ | | | | | | | | | | | | | | | |
| --- | --- | --- | --- | --- | --- | --- | --- | --- | --- | --- | --- | --- | --- | --- | --- |
|  | NH | H_α_ | H_β2_ | H_β3_ | H_γ2_ | H_γ3_ | H_δ2_ | H_δ3_ | | H_ε_ | | | | H_ζ_ | |
| Ac-Arg552 |  | 4.31 (-0.01) | 1.80 | | 1.64 | | 3.17 | | | 7.27 | | | |  | |
| Glu553 | 8.64 | 4.36 (0.07) | 2.03 | | 2.44 | |  | | |  | | | |  | |
| Val554 | 8.29 | 4.08 (0.01) | 2.06 | | 0.89 (Me) | |  | | |  | | | |  | |
| Leu555 | 8.35 | 4.36 (0.02) | 1.64 | | 1.64 | | 0.91 (Me) | | |  | | | |  | |
| Lys556 |  |  |  | |  | |  | | |  | |  | |  | |
| Arg557 |  | 4.31 (0.00) | 1.80 | | 1.64 | | 3.17 | | | 7.31 | | | |  | |
| Glu558 | 8.54 | 4.33 (0.04) | 2.00 | | 2.39 | |  |  | |  | |  | |  | |
| Leu559 | 8.30 | 4.33 (-0.01) | 1.89 | | 1.60 | | 0.89 (Me) | |  | | | | | |  |
| Gly560 | 8.47 | 3.94(-0.05) |  | |  | |  | | |  | | | |  | |
| Leu561 | 8.13 | 4.37 (0.01) | 1.66 | | 1.60 | | 0.89 (Me) | | |  | |  | |  | |
| Ala562 | 8.61 | 4.34 (0.02) | 1.46 (Me) | |  | |  | | |  | | | |  | |
| Glu563 | 8.42 | 4.34 (0.01) | 2.06 | | 2.48 | |  | |  | | | | | |  |
| Ser564 | 8.37 | 4.48 (0.05) | 3.98 | |  | | | | | | | | | | |
| Asp565 | 8.49 | 4.73 (0.09) | 2.86 | |  | |  | | |  | | | |  | |
| Ile566 | 8.11 | 4.21 (0.04) | 1.91; 0.87 (Me) | | 1.17; 1.48 | | 0.87 (Me) | |  | |  | |  | | |
| Ile567 | 8.30 | 4.06 (-0.07) |  | |  | |  | |  | |  | |  | | |
| Asp568 | 8.47 | 4.73 (0.09) | 2.83 | |  | |  | |  | | | | | | |
| Ile569 | 8.19 | 4.38 (-0.04) | 1.87; 0.87 (Me) | | 1.21; 1.48 | | 0.87 (Me) | |  | | | | | | |
| Pro570 |  | 4.36 (-0.05) | 2.10 | | 2.46 | | 3.72;3.90 | |  | | | | | | |
| Gln571 | 8.53 | 4.34 (0.12) | 2.00 | | 2.36 | | 7.01; 7.65 | | |  | | | |  | |
| Leu572 | 8.29 | 4.31 (0.04) | 1.80 | | 1.50 | | 0.84 (Me) | | |  | | | |  | |
| Phe573 | 8.26 | 4.66 (0.00) | 3.00; 3.20 | |  | | 7.22 | | | 7.32 | | | | 7.37 | |
| Lys574 | 8.50 | 4.34 (-0.03) | 1.75 | | 1.47 | |  | | |  | |  | |  | |
| Thr575 | 8.21 | 4.34 (0.06) | 4.25 | | 1.29 (Me) | |  | | |  | | | |  | |
| Glu576 | 8.50 | 4.33 (0.05) | 2.05 | | 2.42 | |  | | |  | | | |  | |
| Arg577 |  | 4.31 (0.01) | 1.80 | | 1.64 | | 3.17 | | | 7.27 | | | |  | |
| Lys578 |  | 4.34* | 1.70* | |  | |  | | |  | |  | |  | |
| Lys579 |  | 4.34* | 1.70* | |  | |  | | |  | |  | |  | |
| Ala580 | 8.50 | 4.34 (-0.05) | 1.46 (Me) | |  | |  | | |  | |  | |  | |
| Thr581-Am | 8.23 | 4.34 (0.02) | 4.25 | | 1.29 (Me) | |  | | |  | |  | |  | |

^a^ The (*) indicates those resonances which could not be unambiguously assigned. For the H_α_ proton column, the values within parenthesis are the conformational shifts (δ_res_-δ_rc_), only shown for those protons unambiguously assigned. For those resonances, which were not unambigously assigned, the conformational shifts were not calculated. The random-coil values for the sequence were obtained from: <https://spin.niddk.nih.gov/bax/nmrserver/Poulsen_rc_CS/>).
